# Evaluation of Fluorine-18-Labeled α1(I)-N-Telopeptide Analogs as Substrate-Based Radiotracers for PET Imaging of Melanoma-Associated Lysyl Oxidase

**DOI:** 10.3389/fchem.2018.00121

**Published:** 2018-04-26

**Authors:** Manuela Kuchar, Christin Neuber, Birgit Belter, Ralf Bergmann, Jens Lenk, Robert Wodtke, Torsten Kniess, Jörg Steinbach, Jens Pietzsch, Reik Löser

**Affiliations:** ^1^Helmholtz-Zentrum Dresden Rossendorf, Institute of Radiopharmaceutical Cancer Research, Dresden, Germany; ^2^Faculty of Chemistry and Food Chemistry, School of Science, Technische Unversität Dresden, Dresden, Germany

**Keywords:** lysyl oxidase, collagen, extracellular matrix, molecular imaging, pharmacokinetics

## Abstract

Accumulating evidence suggests an unequivocal role of lysyl oxidases as key players of tumor progression and metastasis, which renders this enzyme family highly attractive for targeted non-invasive functional imaging of tumors. Considering their function in matrix remodeling, malignant melanoma appears as particularly interesting neoplasia in this respect. For the development of radiotracers that enable PET imaging of the melanoma-associated lysyl oxidase activity, substrates derived from the type I collagen α1 N-telopeptide were labeled with fluorine-18 using *N*-succinimidyl 4-[^18^F]fluorobenzoate ([^18^F]SFB) as prosthetic reagent. With regards to potential crosslinking to tumor-associated collagen *in vivo*, their interaction with triple-helical type I collagen was studied by SPR. A mouse model of human melanoma was established on the basis of the A375 cell line, for which the expression of the oncologically relevant lysyl oxidase isoforms LOX and LOXL2 was demonstrated in Western blot and immunohistochemical experiments. The radiopharmacological profiles of the peptidic radiotracers were evaluated in normal rats and A375 melanoma-bearing mice by *ex vivo* metabolite analysis, whole-body biodistribution studies and dynamic PET imaging. Out of three ^18^F-labeled telopeptide analogs, the one with the most favorable substrate properties has shown favorable tumor uptake and tumor-to-muscle ratio. Lysyl oxidase-mediated tumor uptake was proven by pharmacological inhibition using β-aminopropionitrile and by employing negative-control analogs of impeded or abolished targeting capability. The latter were obtained by substituting the lysine residue by ornithine and norleucine, respectively. Comparing the tumor uptake of the lysine-containing peptide with that of the non-functional analogs indicate the feasibility of lysyl oxidase imaging in melanoma using substrate-based radiotracers.

## Introduction

Malignant melanoma is a highly aggressive and treatment-resistant malignancy of melanocytes, which occurs primarily in fair-skinned populations (Tandler et al., 2012). Melanoma incidence and mortality have been steadily increasing in almost all countries. Referring exemplarily to statistical data from Germany, the incidence rates (mortality rates) of cutaneous melanoma in 2014 were 18.6 (2.9) per 100,000 males and 16.6 (1.7) per 100,000 females, with cutaneous melanoma responsible for about 1.3% of all cancer deaths^1^. It has to be emphasized that melanoma is characterized by high genotypic and phenotypic heterogeneity (Kunz and Hölzel, 2017). This is impressively reflected by the fact that even melanoma cell lines among themselves seem to exhibit different synthesis and secretion pathways for the same protein (Francis et al., 1996).

Lethality of malignant melanoma is almost exclusively due to metastasis from primary sites to non-adjacent organs. In general, metastasis of malignant tumors to distant sites requires a complex interplay of tumor cells, non-malignant cells and the extracellular matrix (ECM). The latter component of the so-called tumor microenvironment is of crucial importance for the metastatic process, as accumulating evidence from recent research suggests (Joyce and Pollard, 2009; Bonnans et al., 2014). The interaction of tumor cells with the ECM mediated by receptors such as integrins underlies invasion of surrounding tissues by tumor cells (Friedl and Alexander, 2011; Lu et al., 2012; Pickup et al., 2014). In addition, malignant and non-malignant cells shape the tumor microenvironment by secreting soluble enzymes that catalyze the posttranslational modifications of matrix proteins. Proteases, which lead to the partial degradation of proteinaceous ECM components, play a pivotal role in that process (Friedl and Wolf, 2008), but also enzymes whose actions result in more subtle structural changes involving the amino acid side chains of proteins are important in that context. Among the latter category of enzymes, lysyl oxidases have been shown to be extraordinarily relevant for the metastasis of solid tumors (Perryman and Erler, 2014).

**Figure 1:**
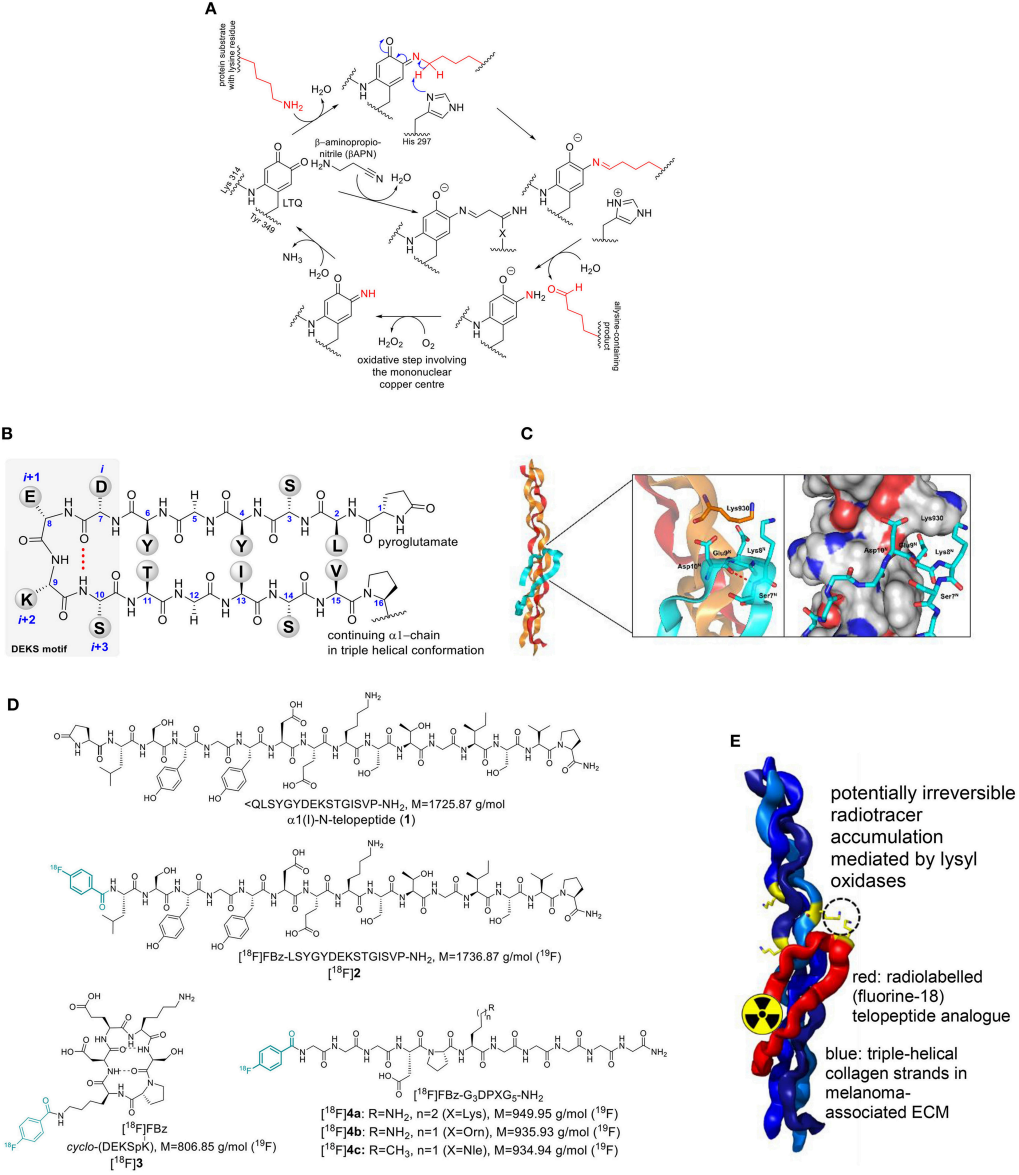
**(A)** Catalytic cycle of lysyl oxidases and inhibition by β-aminopropionitrile (βAPN). LTQ undergoes condensation between one of its carbonyl groups with the ε-amino group of the lysine residue. Deprotonation and subsequent hydrolytic release of the allysine product leads to a 1,2-aminoquinolate intermediate, which is oxidized to a quinone imine by oxygen mediated by the mononuclear copper center under the formation of hydrogen peroxide. Hydrolytic release of ammonia from the quinone imine intermediate regenerates the LTQ cofactor for another catalytic cycle. “X” denotes a nucleophilic residue that has yet to be identified. **(B)** Sequence and secondary structure of the N-terminal telopeptide of the α1 chain of type I collagen in β-hairpin conformation. **(C)** Model of the α1(I)-N-telopeptide docked to the triple-helical receptor region of bovine type I collagen. The α1 chains are shown in ochre, the α2 chain in red and the α1-N-telopeptide in cyan. The β(I)-turn forming DEKS sequence (telopeptide) and Lys930 in (triple helix) are shown in stick representation. Generated by PyMOL (DeLano, W. L. The PyMOL Molecular Graphics System. Version 1.5.0.3 Schrödinger, LLC) using the model provided by Malone et al. (2004) **(D)** Structures of α1(I)-N-telopeptide analogs employed in this study. **(E)** Cartoon-like representation for contrast-generating accumulation of radiolabeled telopeptide analogs by potential lysyl oxidase-mediated crosslinking to triple-helical collagen. Structures are identical to Figure 1C.

Lysyl oxidases (LOs) are a family of copper-dependent amine-oxidases, which catalyze the oxidative deamination of lysine residues in proteins to allysine residues. This transformation is mediated by their unique lysyl tyrosyl quinone (LTQ) cofactor as shown in Figure 1A in mechanistic detail. In humans and other mammals, the LO family is constituted by five proteins, the prototypic lysyl oxidase LOX and the lysyl oxidase-like enzymes LOXL1-4, which show high sequence homology between their C-terminal catalytic domain but differ in the N-terminal regions. All five members are potentially implicated in tumor progression and metastasis (Barker et al., 2012). The best defined and understood function of LOs is oxidative cross-linking of ECM proteins such as collagens and elastin following the generation of reactive allysine residues (Trackman, 2016a). However, their action is not restricted to extracellular protein substrates and LO-mediated posttranslational modification of intracellular proteins seems to be of biological significance also with regards to tumor progression (Iturbide et al., 2015). Via their collagen-crosslinking function, LO activity can lead to increased tensile strength of the ECM (Makris et al., 2015). In turn, mechanical rigidity of tissues can promote tumor progression (Butcher et al., 2009; Wirtz et al., 2011). In a ground-breaking study, LOX has been identified as a key player of hypoxia-induced metastasis (Erler et al., 2006). Furthermore, the involvement of the secreted enzyme in tumor progression has been correlated to enhanced crosslinking of different types of collagen in mouse models of breast cancer (Ng and Brugge, 2009). In particular, this finding was proven to be valid for both the tumor growth at the primary site (Levental et al., 2009) as well as for the formation of the pre-metastatic niche in distant target organs (Erler et al., 2009). Beside the action of LOX, crosslinking of collagen by secreted LOXL2 has been shown to contribute to breast cancer progression by promoting integrin signaling via the focal adhesion kinase (FAK). Both LO isoforms share 48% of sequence identity in their C-terminal catalytic domains while they differ considerably in their N-terminal domains. In contrast to LOX, LOXL2 lacks the propeptide and contains four scavenger receptor cysteine-rich (SRCR) domains (Moon et al., 2014). In addition to mammary tumors, increased expression of LOX and LOXL2 has been shown to drive tumor progression in a variety of different malignancies, as listed in Table 1. With regards to the role of increased LO activity in melanoma, it has been found that the expression of LOX in epithelioid and spindle uveal melanoma was significantly higher in the malignant than in the normal tissue as assessed by immunohistochemical analysis of biopsy samples, whereas no LOXL2 positive staining was detectable. Staining was more intense for the more aggressive epithelioid cell type and increased LOX expression was highly correlated to reduced metastasis-free survival (Abourbih et al., 2010). Treatment of mice bearing tumors derived from the B16F-10 cell line, a syngeneic mouse model of melanoma, with a methanolic extract of the medicinal plant *Vernonia cinerea*, resulted in significantly reduced metastasis to the lung compared to the untreated control. Of note, suppression of metastasis was accompanied by downregulation of the LOX mRNA in the pulmonary tissue (Pratheeshkumar and Kuttan, 2011).

**Table 1:** Selected literature references with regards to involvement of lysyl oxidase isoforms LOX and LOXL2 in various kinds of tumors in addition to breast cancer and melanoma.

**Tumor entity**	**Isoform**	**References**
Lung	LOX	Liu et al., 2014
	LOXL2	Peinado et al., 2008; Peng et al., 2016
Colorectal	LOX	Cox and Erler, 2013
	LOXL2	
Pancreatic	LOX	Miller et al., 2015; Calvé et al., 2016
	LOXL2	Rückert et al., 2010; Miller et al., 2015; Park et al., 2016
Gastric	LOX	Kasashima et al., 2016
	LOXL2	Peng et al., 2009
Prostate	LOX	Lapointe et al., 2004
	LOXL2	Schmidt et al., 2007
Glioma	LOX	Laczko et al., 2007; Mammoto et al., 2013; da Silva et al., 2015
squamous carcinoma of head and neck	LOX	Albinger-Hegyi et al., 2010; Hua et al., 2016
	LOXL2	Martin et al., 2015
Skin	LOX	Mittapalli et al., 2016
	LOXL2	Peinado et al., 2008

The compelling evidence of the involvement of the LO family in tumor progression and metastasis suggests pharmacological inhibition of LOs as a therapeutic option to suppress neoplasia in addition to conventional treatments such as chemo- and radio-therapy. Administration of the potent inhibitor β-aminopropionitrile (βAPN) to tumor-bearing mice led to reduced malignant progression in preclinical *in vivo* models of various cancers, particularly with regards to attenuation of metastasis (Barker et al., 2012; Pickup et al., 2013; Cox et al., 2016). Considering the upregulation of LOX during tumor hypoxia, reductively activatable prodrugs of βAPN have been developed, which were shown to be capable of inhibiting the invasion of MDA-MB-231 breast tumor cells under hypoxic conditions *in vitro* (Granchi et al., 2009). Even though βAPN is selective in inhibiting LOs over other amine oxidases, the agent does not differentiate between individual LO family members (Tang et al., 1989; Moon et al., 2014). For selective targeting of LOX and LOXL2, antibodies have been used (Barker et al., 2012). The application of a LOX-directed antibody, which is capable of inhibiting enzymatic function, in a mouse model of breast cancer resulted in decreased number of pre-metastatic osteolytic lesions (Cox et al., 2015). Antibody-based targeting of LOXL2 has been shown to be effective in reducing lung metastasis in mouse xenografts derived from the BGC823 gastric cancer cell line (Peng et al., 2009). Another LOXL2-directed antibody, AB0023, which binds allosterically to the fourth SRCR domain located at the N-terminus of the protein, potently inhibits catalytic activity in a partial, non-competitive manner (Rodriguez et al., 2010). Administration of AB0023 to mice bearing tumors derived from the SKOV-3 ovarian carcinoma cell line inhibited angiogenesis in these neoplasms (Zaffryar-Eilot et al., 2013).

The crucial role of LOs in tumor progression, the prognostic value of their expression and the promising potential of targeting LOs in the context of antimetastatic therapy encourages the development of agents that are capable of imaging these enzymes *in vivo*. For this purpose, positron emission tomography (PET) represents the most advantageous imaging modality in terms of sensitivity, penetration depth and quantification (James and Gambhir, 2012). This method requires the application of molecular probes labeled with positron-emitting radionuclides, among which fluorine-18 represents the most optimal one due to its advantageous particle energy (E_max_ = 635 keV) and high branching ratio for positron emission of 97% (Jacobson et al., 2015). To the best of our knowledge, no studies toward the development of LO-directed probes for *in vivo* imaging have been reported, apart from the efforts by our group (Kuchar et al., 2013; Wuest et al., 2015).

The peptidic LO substrate [^18^F]**4a**, which has been developed within this study, was independently evaluated in a syngeneic mouse model of breast cancer (Wuest et al., 2015). Herein we explored the opportunity of imaging the melanoma-associated LO activity by substrate-based radiotracers. This includes probe design and synthesis, establishment of an appropriate animal model and the radiopharmacological *in vivo* characterization of compound [^18^F]**4a** in comparison to other substrate-based radiotracers and to provide extended proof of LO targeting by employing non-functional analogs. The results should provide proof of concept toward the general applicability of substrate-based radiotracers for imaging of the tumor-associated LO activity *in vivo*.

## Results and discussion

### Design of the radiolabeled probes

The availability of small molecule-based inhibitors of LOs that bear pharmacological value and are potentially suitable for molecular imaging is very limited. β-Aminopropionitrile (βAPN) seems to be the most potent inhibitor for this enzyme family (Šebela and Sayre, 2009; Trackman, 2016b; Figure 1A). Considering its very low molecular weight, βAPN is hardly amenable to labeling with fluorine-18. Taking into consideration the function of LOs in cross-linking of connective tissue proteins, which occurs mainly by Schiff base formation between the allysine-containing products and the unmodified lysine side chains, a radiotracer design starting from substrates was envisaged. Protein crosslinking by Schiff base formation does not involve enzymatic catalysis (Trackman, 2016a). Therefore, radiolabeled lysine-containing peptidic substrates should spontaneously react with lysine residues on extracellular tumor-associated proteins after conversion to allysine by lysyl oxidase. In turn, this will lead to radiotracer accumulation in regions of high lysyl oxidase activity (Figure 1E). Irrespective of such a scenario, substrate-based radiotracers can be suitable for imaging of enzymes *in vivo* even in the absence of irreversible product trapping, as demonstrated for PET imaging of histone deacetylases (Seo et al., 2013; Bonomi et al., 2015).

One of the most abundant ECM proteins is type I collagen. Lysyl oxidase-mediated crosslinking in this collagen type occurs predominantly between allysine residues in the telopeptides and lysine or 5-hydroxylysine side chains in the triple-helical regions (Eyre and Wu, 2005). The crosslinking process is especially well-understood for the N-terminal telopeptide of the α1(I) chain (α1(I)-N-telopeptide). There, the lysine residue undergoes oxidation to act as aldehyde donor toward a (hydroxy)lysine residue located at the α1 chain in the C-terminal region of the triple-helical portion (position 930) of another type I collagen molecule. The structure of the α1(I)-N-telopeptide is shown in Figure 1B. It has been proposed that the collagen-bound peptide adopts a hairpin-like conformation when bound to the triple-helical receptor region (Helseth et al., 1979). Notably, the central sequence Asp-Glu-Lys-Ser (DEKS) forms a β-turn in that conformation (George et al., 1999; Malone et al., 2004; Figure 1C). Therefore, we intended to use α1(I)-N-telopeptide analogs as substrate-based radiotracers for lysyl oxidase imaging. To enable labeling with the PET nuclide fluorine-18, the N-terminal pyroglutamate in **1** was replaced by a 4-fluorobenzoyl residue leading to peptide **2** (Figure 1D). Furthermore, a cyclic hexapeptide (**3**) which conformationally restricts the DEKS motif in the βI-turn as relevant for complexing with collagen has been designed and synthesized (Wodtke et al., 2015). Various lysine-containing oligopeptides derived from the α1(I)-N-telopeptide have been analyzed with regards to their kinetics of LOX-catalyzed deamination, which has revealed the undecapeptide Ac-G_5_DPKG_3_-NH_2_ as the most suitable substrate (Nagan and Kagan, 1994). Replacement of its N-terminal acetyl group by 4-fluorobenzoyl for the purpose of ^18^F-labeling led to peptide **4a**. As outlined below, [^18^F]**4a** was investigated in more detail toward its suitability as imaging agent for LOX. Therefore, derivatives of peptide **4a** in which the lysine residue was substituted by ornithine (peptide **4b**) and norleucine (peptide **4c**) were synthesized and labeled with fluorine-18 to serve as negative-control agents of diminished (Trackman and Kagan, 1979) or abolished LOX-targeting capability, respectively. Labeling of the telopeptide analogs **2** and **4a** with fluorine-18 has been separately investigated within a previous radiochemical study (Kuchar et al., 2012). The elaborated method was adapted for ^18^F-labeling of cyclohexapeptide **3** and peptides **4b** and **4c**.

### Interaction of the telopeptide analogs with type I atelocollagen

As the telopeptide-collagen interaction is determining lysyl oxidase-catalyzed crosslinking, the binding affinity of the α1(I)-N-telopeptide and the derived analogs shown in Figure 1D to type I atelocollagen (i.e., pepsin-treated bovine type I collagen) was investigated by surface plasmon resonance (SPR). Due to binding of the cyclohexapeptide **3** to the unmodified dextran matrix of the SPR chip, it was not possible to characterize its interaction with collagen. The sensorgrams for peptides **1**, **2**, and **4a** are shown in Figure S1 in Supplementary Material and the calculated dissociation constants are included in Table 2. Surprisingly, the authentic isolated α1(I)-N-telopeptide (**1**) exhibited the lowest affinity toward type I atelocollagen with a determined *K*_D_ value in the single-digit millimolar range. Unexpectedly, replacement of the N-terminal pyroglutamate by the 4-fluorobenzoyl group (peptide **2**) lowers the *K*_D_ value by approximately two orders of magnitude. Worth of note, the DPK-containing undecapeptide **4**, which shares the Lys and Asp residue in positions *i* and *i*-2, respectively, with the other telopeptide analogs but otherwise differs significantly in its structure, displays a binding affinity to atelocollagen that is in between those of peptides **1** and **2**.

**Table 2:** Rate constants for the interaction of telopeptide derivatives **1**, **2**, and **4a** with immobilized bovine atelocollagen derived from surface plasmon resonance binding experiments according to a two-step binding model.

**Peptide**	***k*_a1_ (M^−1^ s^−1^)**	***k*_a2_ (s^−1^)**	***k*_d1_ (s^−1^)**	***k*_d2_ (s^−1^)**	***K*_D_ (mM)**	***k*_off_ (s^−1^)**	***k*_on_ (M^−1^ s^−1^)**	***n***
**1**	1.23 × 10^2^	5.32 × 10^−3^	7.50 × 10^−1^	2.94 × 10^−3^	2.17	2.91 × 10^−3^	1.34	5
	2.20–434	(1.01–8.18) × 10^−3^	5.89 × 10^−2^-1.85	(2.33–3.91) × 10^−3^				
**2**	57.4	1.41 × 10^−2^	6.31 × 10^−2^	1.09 × 10^−3^	7.88 × 10^−2^	8.79 × 10^−4^	11.2	5
	50.2-69.8	(1.29–1.70) × 10^−2^	(5.69–6.98) × 10^−2^	(0.87-1.20) × 10^−3^				
**4a**	1.88 × 10^2^	4.94 × 10^−3^	2.68 × 10^−1^	4.11 × 10^−3^	6.47 × 10^−1^	3.98 × 10^−3^	6.15	5
	(0.64–2.97) × 10^2^	(3.72–6.85) × 10^−3^	5.41 × 10^−2^-4.84 × 10^−1^	(3.82-4.43) × 10^−3^				

*Given are the mean values, the range of values is shown below each mean value. Dissociation constants (K_D_) and global rate constants k_off_ and k_on_ describing the dissociation and association of/to the final complex, respectively, were calculated as described in Materials and Methods section. **1**, pGlu-LSYGYDEKSTGISVP-NH_2_; **2**, FBz-LSYGYDEKSTGISVP-NH_2_; **4a**, FBz-GGGDPKGGGGG-NH_2_. Representative sensograms for each compound are shown in Supplementary Material (Figure S1)*.

The *K*_D_ values stated in Table 2 allow for concluding that the non-covalent interaction between the N-telopeptide derivatives and their receptor region on triple-helical collagen is of rather weak character. It should be emphasized that the data shown here, to the best of our knowledge, provide for the first time insight on the strength of the telopeptide-collagen interaction. Considering the critical role of the telopeptides in crosslink formation, the obtained results suggest that additional interactions between the triple-helical domains of opposing collagen strands exist that preorganize the collagen molecules in a way that facilitates binding of the telopeptides to their triple-helical receptor regions so that lysine residues are brought into proximity for lysyl oxidase-catalyzed crosslinking. This assumption is supported by the finding that self-assembly of type I collagen strands into fibrils can occur even in the presence of truncated telopeptides (Kuznetsova and Leikin, 1999). Therefore, the obtained data indicate that crosslinking of isolated telopeptides to triple-helical collagen strands is unlikely, due to their weak interaction.

### Expression analysis for lysyl oxidase isoforms and development of mouse tumor xenograft models

**Figure 2:**
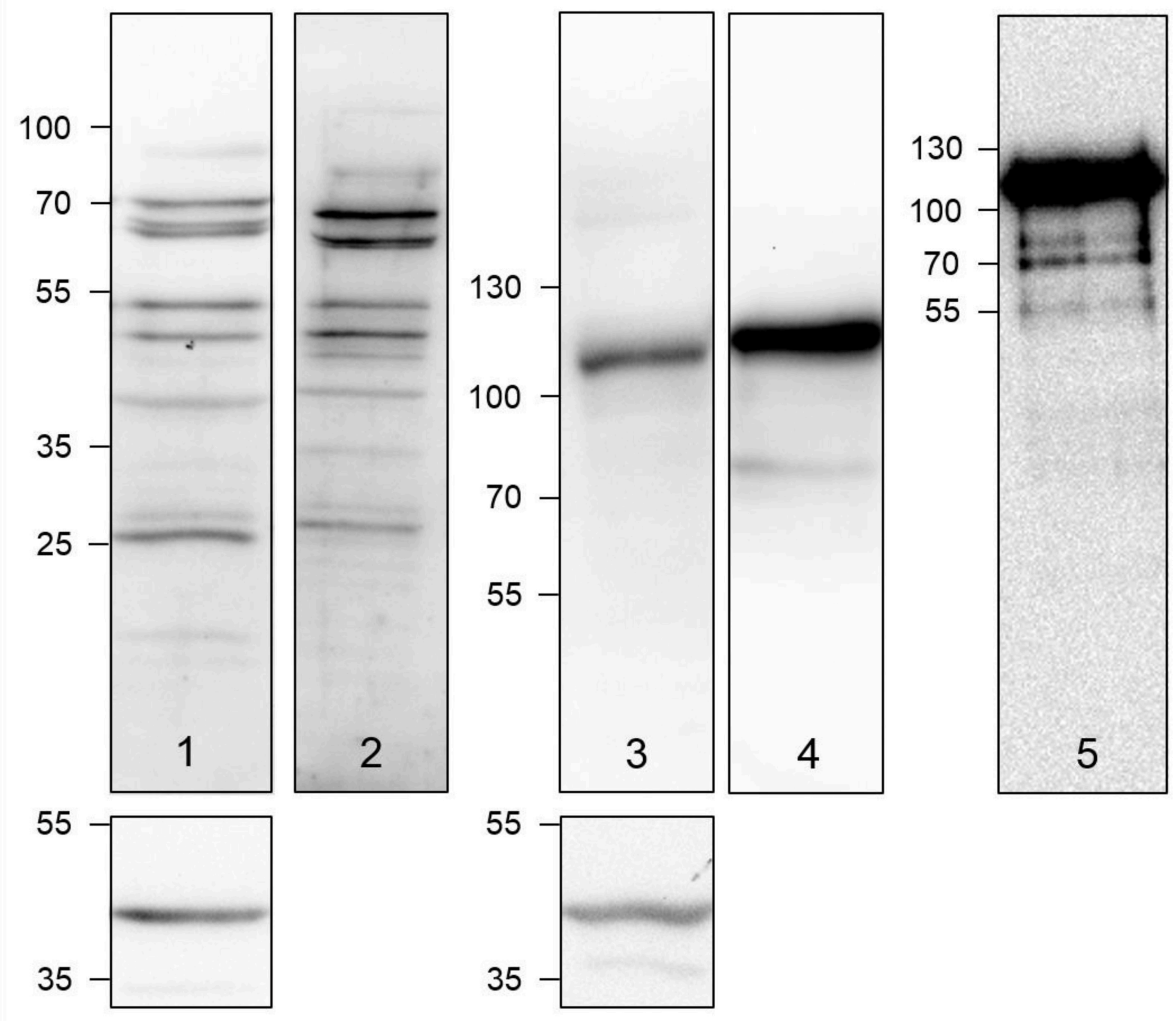
Detection of LOX (lanes 1 and 2) and LOXL2 (lanes 3 and 4) in A375 cell lysates (lanes 1 and 3) and cell culture supernatants (lanes 2 and 4) by Western blot. Lane 5 shows the Western blot of a commercially obtained authentic LOXL2 sample. The sections below lanes 1 and 3 show the Western blots for the actin loading control. Semiquantitative expression data can be found in Figure S2 and Table S1 in Supplementary Material.

As A375 cells represent a well-characterized metastatic melanoma cell line (Herwig et al., 2016a,b), these cells were investigated for the presence of the oncologically relevant lysl oxidase homologs LOX and LOXL2 at the protein level. As both intra- and extra-cellular functions were assigned to both enzymes in the context of tumor progression (Barker et al., 2012), cell lysates, and culture supernatants were analyzed by Western blots (Figure 2).

Using a LOX-specific primary antibody, LOX-positive protein bands were detectable in the A375 cell lysate and with even higher intensity in the supernatants. The obtained pattern of LOX species includes the double bands in the range of 70 kDa, below 55 kDa and in the range of 25–30 kDa. The phenomenon of multiple LOX species appearing in Western blots has been reported (Trackman et al., 1991; Kagan et al., 1995; Roy et al., 1996). The bands in the range below 55 kDa can be assigned to the LOX proenzyme, whose molar mass is ranging between 45 and 50 kDa, depending on the glycosylation status. Therefore, the two distinct bands in that mass range can be attributed to glycosylation variants (Trackman et al., 1992; Grimsby et al., 2010). The bands representing the mature LOX protein appear at about 30 kDa, as expected. The molar mass of the mature enzyme can range between 28, 29, 32, and 40 kDa due to the presence of multiple sites of proteolysis for removal of the LOX propeptide, whose total molar mass is 18 kDa (Kagan et al., 1995; Panchenko et al., 1996). The appearance of slowly migrating LOX antibody-detectable species around 70 kDa cannot be conclusively explained. The reason might be the aggregation of mature LOX species to dimers as it is known that LOX is prone to aggregation, which has been attributed to its flexible tyrosine-rich N-terminal tail (Kagan and Ryvkin, 2011).

Detection of LOXL2 in the A375 cell lysates and culture supernatant by Western blot was dominated by a band at 115 kDa, while a band at 85 kDa, the expected molar mass for LOXL2 (Payne et al., 2007), was only visible for the supernatant. Western blot of commercially available LOXL2 verified that the 115 kDa band represents active LOXL2. In contrast to LOX, LOXL2 lacks a propeptide sequence but contains three potential glycosylation sites, for which Asn 455 and Asn 644 have been confirmed to undergo N-glycosylation (Moon et al., 2014). Therefore, the 115 kDa band can be probably assigned to glycosylated LOXL2.

**Figure 3:**
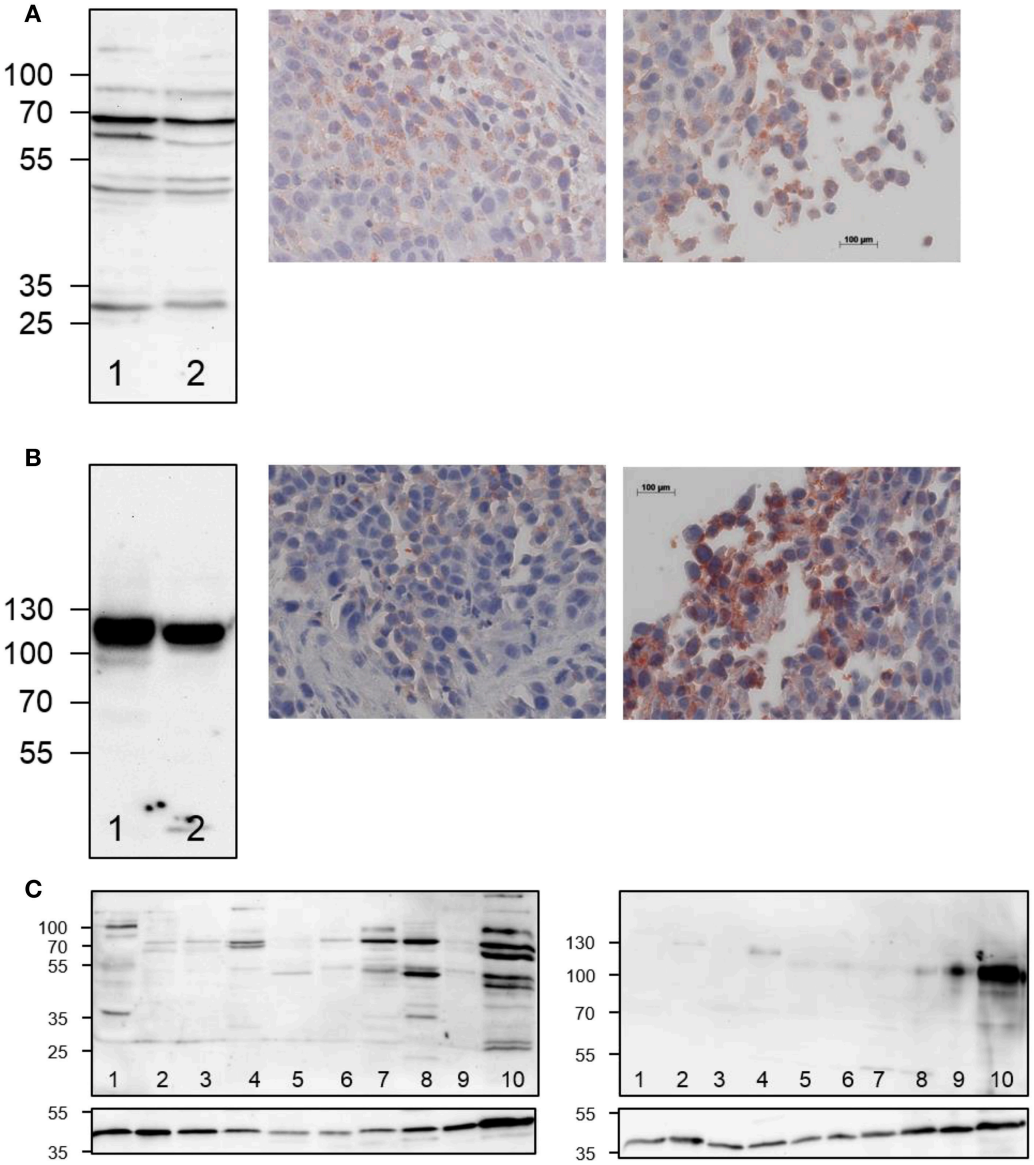
*Ex vivo* detection of LOX and LOXL2 expression in A375 tumor-bearing NMRI (nu/nu) mice. **(A)** Western blot analysis (left) of LOX expression in A375 cells (lane 1) and in tumor tissue homogenate (lane 2) and immunohistochemical staining of LOX in tumor sections (right images), **(B)** Western blot analysis (left) of LOXL2 expression in A375 cells (lane 1) and in tumor tissue homogenate (lane 2) and immunohistochemical staining of LOXL2 in tumor sections (right images). Photographs in the middle represent inner tumor areas whereas photographs to the right show regions at the rim of the tumor. Images that show the entire tumor area are included in Supplementary Material (Figure S3). **(C)** Western blot analysis for LOX and LOXL2 expression in normal vs. tumor tissue (20 μg of protein per lane), 1: bladder, 2: uterus, 3: pancreas, 4: kidney, 5: ear, 6: ovary, 7: liver, 8: heart, 9: lung, 10: A375 tumor. The sections below the actual blots show the Western blots for the actin loading control.

As the expression of the oncologically most important LO family members LOX and LOXL2 in human A375 cells was confirmed, a melanoma xenograft model was established (Mamat et al., 2012) as lined out in the Materials and Methods section. Western blot analysis of the tumor tissue indicated the expression of LOX and LOXL2, which was slightly increased compared to cells cultured *in vitro* (Figure 3A). As obvious from Figure 3B, this result is confirmed by immunohistochemical detection of both enzymes in tumor sections. Notably, staining for both enzymes was stronger at the rim of tumor section than in the center. In addition to tumor tissue, homogenates of various other organs were investigated for the presence of LOX and LOXL2 by Western blot. The results are shown in Figure 3C and indicate that the expression of both LO family members is most pronounced in tumor tissue. However, significant LOX expression was also detectable in heart, liver, kidney, and bladder tissue. In contrast, LOXL2 expression was much more intense in tumor compared to normal tissue. LOXL2 positive bands were clearly visible in lung, heart, and kidney tissue whereas the bands for the other organs were very weak. Therefore, it can be concluded that both LOX and LOXL2 are expressed in the tumor tissue, which renders the established melanoma xenograft model suitable for evaluation of the radiolabeled probes.

### Radiopharmacological characterization

#### Metabolic stability and *ex vivo* biodistribution in normal wistar rats

To obtain general insight into their pharmacokinetic behavior, the fate of the radiolabeled peptides was studied by *ex vivo* methods in healthy Wistar rats including assessment of metabolic stability and whole-body biodistribution.

**Figure 4:**
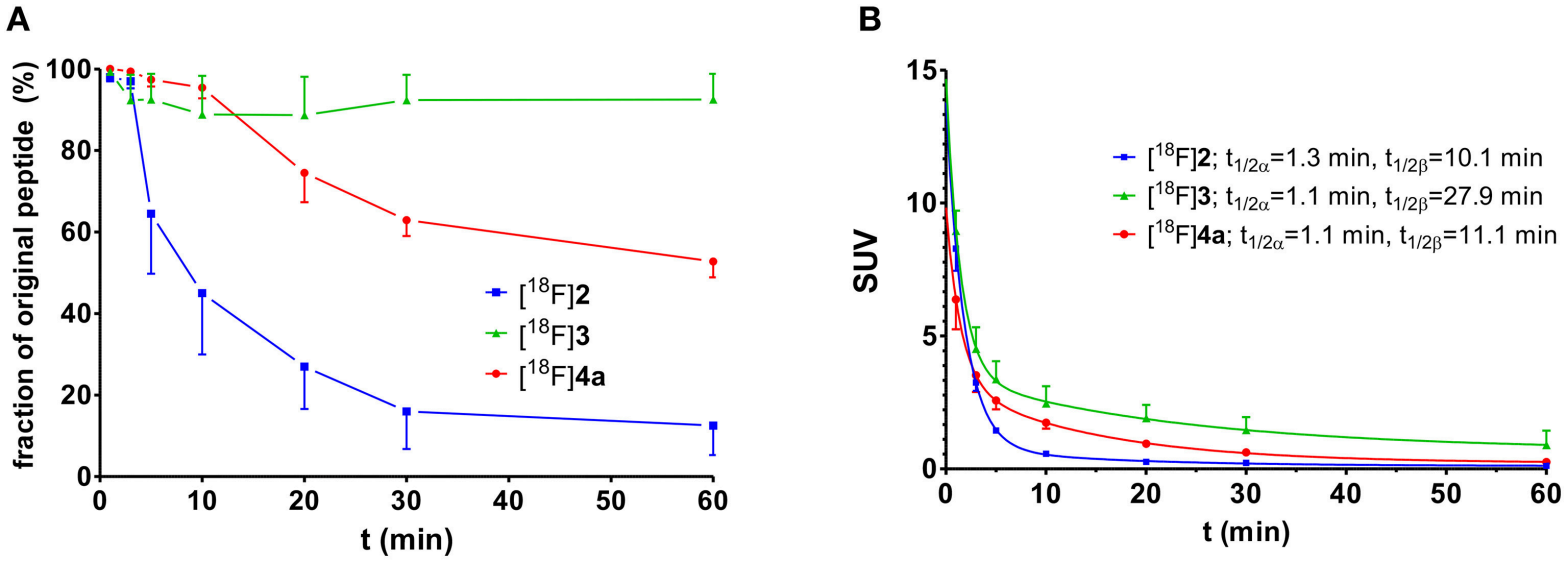
Investigation of metabolic stability and arterial blood clearance of [^18^F]**2** (*n* = 3), [^18^F]**3** (*n* = 3), and [^18^F]**4a** (*n* = 4) in healthy male Wistar rats. **(A)** Time course of metabolic transformation as derived from radio-HPLC analysis of withdrawn blood samples. **(B)** Time curves for arterial blood clearance as calculated from activity measurements of blood samples and fractions of original peptide. t_1/2α_ and t_1/2β_ signify the half-lives of the distribution (α) and elimination phase (β) according to a two-compartment model, respectively.

The metabolic stability of the radiolabeled peptides [^18^F]**2**, [^18^F]**3**, and [^18^F]**4a** was studied in normal Wistar rats. Analysis of the blood fractions that were processed according to standard protocols (see section Materials and Methods) indicated that no accumulation in blood cells or binding to plasma proteins occurs. Radio-HPLC of the deproteinised plasma samples provided insight into the *in vivo* stability of the tracer peptides. With less than 40% of intact peptide remaining after 20 min, the ^18^F-fluorobenzoylated N-telopeptide ([^18^F]**2**) was least stable among the three compounds (Figure 4A). No degradation was detectable for cyclohexapeptide [^18^F]**3** over the investigated time range, as expected from its stability toward trypsin *in vitro* (Wodtke et al., 2015). Peptide [^18^F]**4a** containing the DPK motif underwent slow degradation *in vivo* with ~50% of original compound remaining after 60 min p.i. Therefore, peptides [^18^F]**3** and [^18^F]**4a** seem to be most suitable as radiotracers with regards to metabolic stability. However, considering the rapid blood clearance of all peptides, the telopeptide derivative [^18^F]**2** was not excluded from further investigations at this stage.

The clearance of all three ^18^F-labeled peptides from rat arterial blood is shown in Figure 4B. Obviously, the distribution half-lives t_1/2α_ are very similar and short, which suggests their rapid distribution over the extracellular fluid, which is in accordance with their hydrophilic character. According to its high metabolic stability, the cyclohexapeptide [^18^F]**3** exhibits the longest elimination half-life t_1/2β_ of all three peptides. The elimination half-life of [^18^F]**4a** is in the same range as that of [^18^F]**2**, despite its significantly higher metabolic stability.

**Figure 5:**
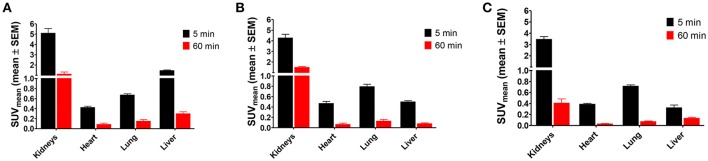
*Ex vivo* biodistribution of ^18^F-labeled peptides **2**, **3**, and **4a** in selected organs of male healthy Wistar rats. **(A)** [^18^F]**2** (*n* = 12); **(B)** [^18^F]**3** (*n* = 12); **(C)** [^18^F]**4a** (*n* = 8).

The biodistribution of the radiolabeled peptides [^18^F]**2**, [^18^F]**3**, and [^18^F]**4a** was investigated in healthy Wistar rats after 5 and 60 min p.i. Diagrams displaying the activity uptake in selected organs are shown in Figure 5, results for all organs can be found in Supplementary Material (Figures S7, S8). High uptake of all three peptides is detectable for the kidneys in the perfusion phase (5 min p.i.), while the SUV decreases below 2 after 60 min, which indicates no accumulation in that organ. The data suggest that renal elimination predominates for all three peptides. For [^18^F]**2** a significantly increased SUV was observed for the liver in the perfusion phase. This result in combination with its low metabolic stability suggests the partial formation of lipophilic radiometabolites which undergo hepatobiliary elimination. Uptake in heart and lungs was determined by perfusion as indicated by the large differences in SUV after 5 and 60 min p.i. Apart from the kidneys and the liver no increased SUV was observed in any other organ, tissue or gland. All three radiolabeled peptides show favorable biodistribution patterns with regards to their use as radiotracers, as no activity accumulation in organs and tissues was observed. Therefore, all three compounds were judged suitable for further radiopharmacological investigations in A375 melanoma-bearing NMRI (*nu*/*nu*) mice.

#### Evaluation in the A375 melanoma xenograft model

**Figure 6:**
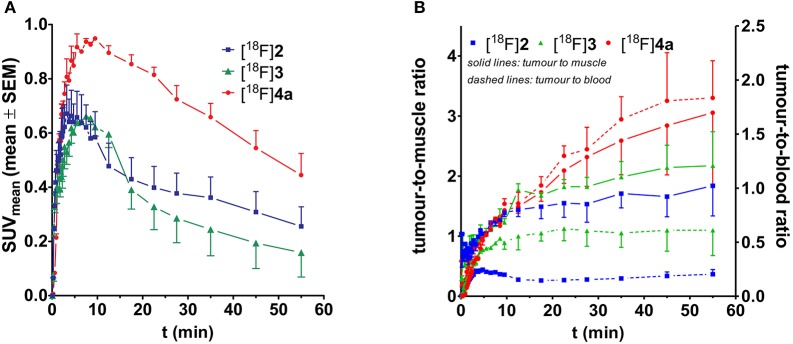
Time-activity curves for the tumor region of the peptidic radiotracers [^18^F]**2** (*n* = 2), [^18^F]**3** (*n* = 2), and [^18^F]**4a** (*n* = 4). **(A)** Time course considering the absolute activity. **(B)** Time course of the tumor-to-muscle and tumor-to-blood ratios.

The time-resolved spatial distribution of [^18^F]**2**, [^18^F]**3**, and [^18^F]**4a** was studied with PET in NMRI (*nu*/*nu*) mice bearing tumor xenografts derived from the A375 cell line. Retrieval of time-activity curves for tumor as regions of interest indicated that the uptake of [^18^F]**4a** reaches significantly greater values than peptides [^18^F]**2** and [^18^F]**3** (Figure 6A). Furthermore, both the tumor-to-muscle and tumor-blood-ratios are greatest for peptide [^18^F]**4a** (Figure 6B). The maximum SUV and time course of the tumor uptake of the DEKS motif-containing telopeptide analogs [^18^F]**2** and [^18^F]**3** are similar. Accordingly, they exhibit similar tumor-to-muscle ratios. In contrast, the tumor-to-blood ratios for cylohexapeptide [^18^F]**3** are higher than that for the linear peptide [^18^F]**2**, which is in line with the greater β phase half-life for blood elimination of [^18^F]**3** observed in rats. Taken together, the tumor-to-muscle ratio reaching values close to 3 combined with absolute SUVs close to 1 and sufficient metabolic stability of the radiotracer suggest a potential target-mediated tumor uptake for the DPK-containing LOX substrate [^18^F]**4a**.

**Figure 7:**
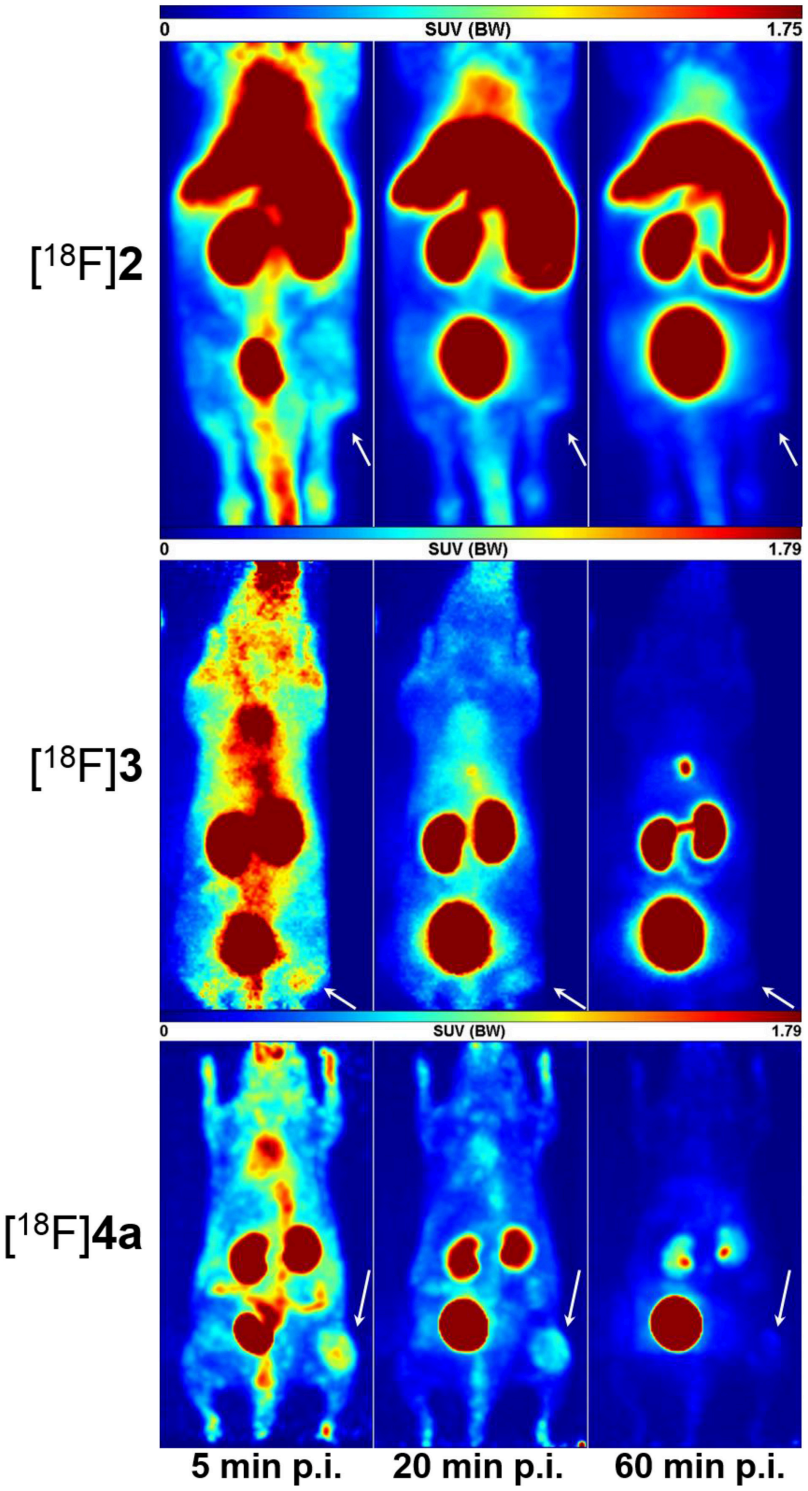
Small animal PET images (maximum intensity projections) of A375 tumor-bearing NMRI (nu/nu) mice depicting the distribution of [^18^F]**2**, [^18^F]**3**, and [^18^F]**4a** each after 5, 20, and 60 min p. i. The location of the tumor tissue is indicated by an arrow.

Static PET images (adjusted maximum intensity projections) for 5, 20, and 60 min p.i. show the spatial distribution of [^18^F]**2**, [^18^F]**3**, and [^18^F]**4a** in the melanoma-bearing animals (Figure 7). The observed activity distribution reflects the pharmacokinetic properties of each peptide. Accordingly, telopeptide [^18^F]**2** shows significant enrichment in the liver and the intestine because of its partial hepatobiliary clearance. PET images obtained with the cyclohexapeptide [^18^F]**3** for 5 min p.i. show enhanced activity uptake in peripheral tissues compared to the other ^18^F-labeled peptides, which is in accordance with its lower clearance rate. As expected from its pharmacokinetic characterisation in rats, the highest uptake of [^18^F]**4a** occurs in the kidneys and the bladder. Comparing the PET images of all three peptides at 20 min p.i., the tumor region can be most suitably distinguished from the surrounding tissue in the case of [^18^F]**4a**.

**Figure 8:**
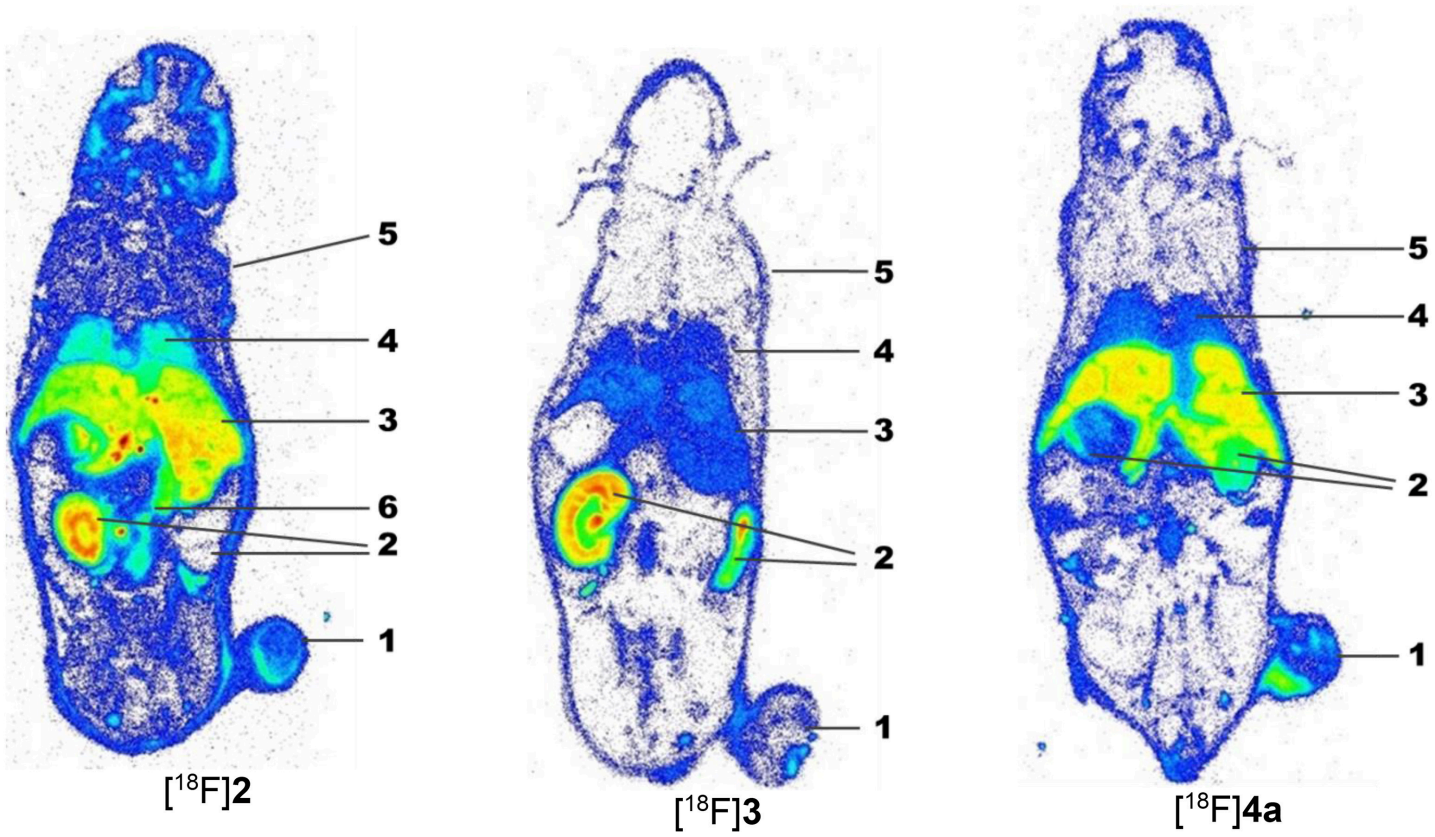
*Ex vivo* whole-body radioluminographs fort the peptidic radiotracers [^18^F]**2**, [^18^F]**3**, and [^18^F]**4a** in A375 tumor-bearing NMRI (*nu/nu*) mice. Animals were sacrificed 60 min after radiotracer injection. The bladder was removed before image preparation. For each compound, one whole-body section in the plane of the maximum dimension of the tumor is shown. 1: A375 tumor, 2: kidneys, 3: liver, 4: lung, 5: skin, 6: intestine.

For complementation of the PET studies, whole-body radioluminographs were prepared for all three peptides at 60 min p.i. The images are shown in Figure 8 and indicate the most obvious tumor uptake for compound [^18^F]**4a**. Activity accumulation for [^18^F]**2** in the tumor region was less pronounced than for [^18^F]**4a**. The cyclohexapeptide [^18^F]**3** showed very low enrichment in the neoplastic tissue. Activity accumulation in other organs is mainly influenced by the elimination pathways of the particular radiotracer. For all three compounds, the bladder was removed from all sections prior to exposure to prevent image bleeding to other regions in close proximity. While the majority of activity accumulated in the kidneys for [^18^F]**4a**, [^18^F]**2**-derived activity appeared in liver and the kidneys to a similar extent. Very low activity uptake in the liver compared to kidney is seen for [^18^F]**3**, which is in accordance with a rapid renal elimination as concluded from the studies discussed above. Notably, for all three peptides, activity enrichment at the margins of the animal bodies was more pronounced than in the carcass, which suggests increased uptake in the skin. This might be indicative of LO targeting since regeneration of the skin requires the turnover of collagen and other elastic fibers. The association of LOX and other LO isoforms with the ECM of the skin has been demonstrated (Romero-Chapman et al., 1991; Noblesse et al., 2004).

#### Proof of LO-mediated tumor targeting

**Figure 9:**
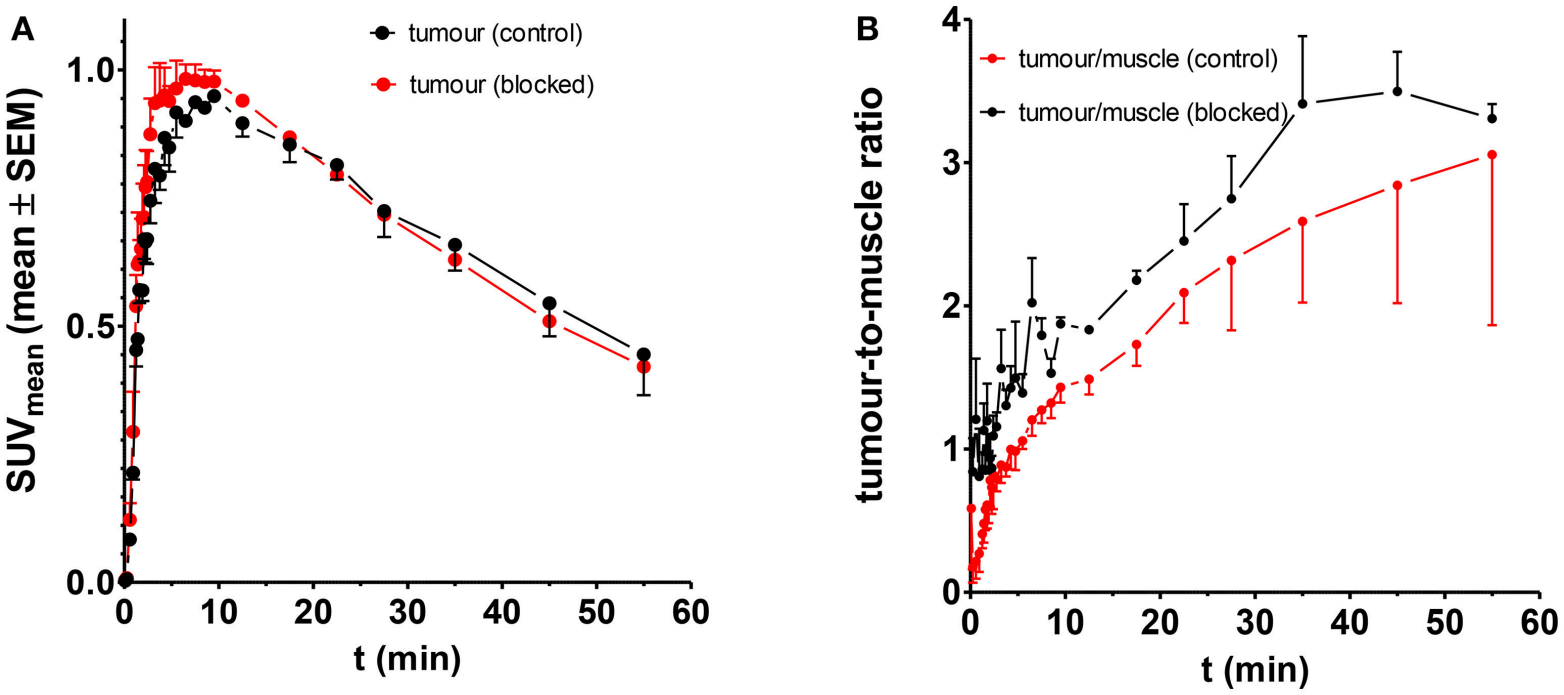
Influence of βAPN on the uptake of [^18^F]**4a** in the tumor tissue in A375 tumor-bearing NMRI (*nu/nu*) mice; *n* = 4 and 2 for control and treated animals, respectively. **(A)** Absolute uptake as quantified in SUV, **(B)** Tumour-to-muscle ratio.

Based on the observations mentioned above that indicated a LO-mediated uptake of [^18^F]**4a** in the A375 melanoma xenografts, further experiments were performed in order to prove LO targeting by this radiotracer. Initial attempts aimed at reducing the uptake of [^18^F]**4a** in the tumor region by pharmacological inhibition of LOs. To this end, the potent LO inhibitor βAPN was administered 4 h before radiotracer injection at a dose of 100 mg/kg. The time-activity curve for tumor tissue as region of interest derived from the dynamic PET imaging investigations are displayed in Figure 9A. As shown, βAPN treatment resulted in a slight increase of radiotracer uptake in the tumor tissue (Figure 9B). In addition, the maximum is slightly shifted toward earlier time points. The increased tumor uptake is accompanied by an increased tumor-to-muscle ratio. Interestingly, uptake in the muscle region was even more increased in the presence of βAPN. Even though such change of radiotracer uptake under pharmacological target inhibition was unexpected, increased tracer uptake under pharmacological blockade was observed occasionally and has been mainly explained with the occupancy of non-saturable binding sites by the blocking agent, which in turn results in increased availability of radiotracer for the specific target sites (Eastman et al., 1992; de Vries et al., 2008; Löser et al., 2013). Moreover, considering the abundant expression of LOX in non-tumor tissue, particularly in well perfused organs such as heart, liver and kidney (Figure 3C), inhibition by βAPN in those tissues might result in increased availability of [^18^F]**4a** for uptake in the tumor. Furthermore, one has to consider that the radiotracer-target interaction resembles in the present case an enzyme-substrate interaction. This means that upon interaction with its target LO [^18^F]**4a** undergoes chemical transformation to the allysine-containing product, which is no longer capable of binding to the target. Therefore, inhibition of LOs by βAPN treatment leads to reduced interaction of [^18^F]**4a** with its target but also reduced turnover of the radiotracer and, consequently, increased blood levels of remaining original [^18^F]**4a** compared to the absence of βAPN. Hence, the increased blood level of remaining original [^18^F]**4a** may lead to increased tumor uptake in the presence of βAPN. This finding is in contrast to observations in a breast tumor mouse model, in which administration of the same LO inhibitor resulted in attenuated tumor uptake of [^18^F]**4a** (Wuest et al., 2015). This discrepancy can be explained with the different biological origin of the tumor tissue. Whereas herein a melanoma xenograft model is used, Wuest et al. were using a syngeneic model derived from the murine mammary carcinoma EMT-6 cell line. In addition, the anatomical localization (right leg vs. left shoulder) is different. Therefore, improved blood supply of the tumor tissue in the syngeneic model is assumed to lead to higher tumor uptake of βAPN and distinctly higher inhibition of tumor-associated LOs compared to the xenograft model. In conclusion, treatment with βAPN leads to increased uptake of [^18^F]**4a** in target-expressing A375 xenograft tissue, which can be interpreted toward LO-mediated tumor uptake. However, the observed effects are rather ambiguous and request additional proof for target-mediated radiotracer uptake.

**Figure 10:**
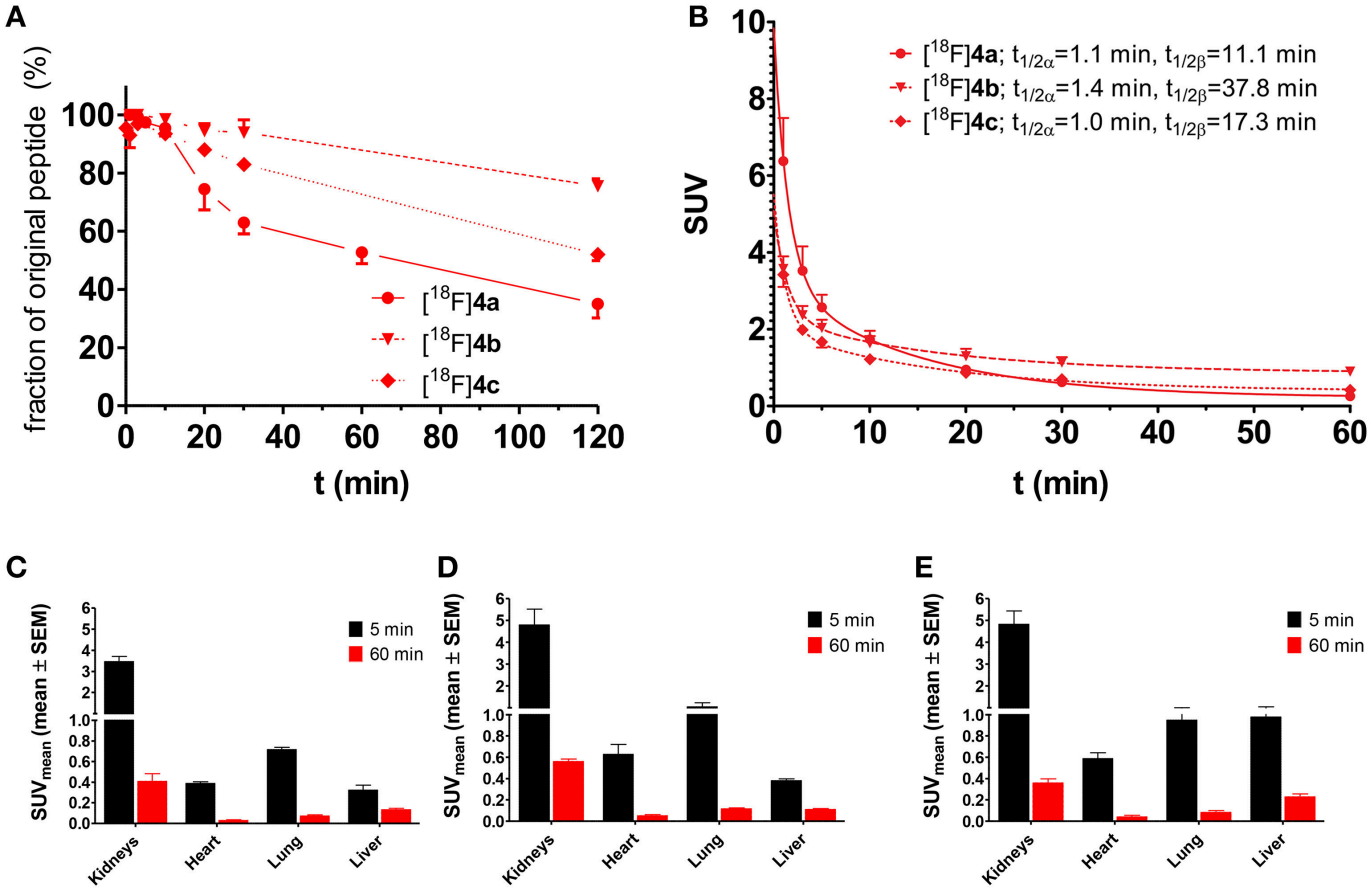
Investigation of the pharmacokinetic properties of [^18^F]**4b** and [^18^F]**4c** *ex vivo* in healthy male Wistar rats. For comparison, results of [^18^F]**4a** have been included (identical data as in Figures 4, 5, respectively). **(A,B)** Metabolic stability and arterial blood clearance, *n* = 2 for [^18^F]**4b** and [^18^F]**4c** each; **(A)** Time course of metabolic transformation as derived from radio-HPLC analysis of withdrawn blood samples. **(B)** Time curves for arterial blood clearance as calculated from activity measurements of blood samples and metabolite fractions. t_1/2α_ and t_1/2β_ signify the half-lives of the distribution and elimination phase according to a two-compartment model, respectively. **(C–E)** *Ex vivo* biodistribution of [^18^F]**4a**–**c** in selected organs of male healthy Wistar rats. **(C)** [^18^F]**4a** (*n* = 8); **(D)** [^18^F]**4b** (*n* = 4); **(E)** [^18^F]**4c** (*n* = 4).

Another approach for proving the target-mediated uptake of radiotracers is the use of analogs, which are structurally similar to the tracer of interest and therefore exhibit comparable pharmacokinetic properties and biodistribution patterns but show impeded or abolished interaction with the target (Haubner et al., 1999). To apply such an approach to the LO substrate [^18^F]**4a**, its lysine residue was replaced by ornithine and norleucine, leading to [^18^F]**4b** and [^18^F]**4c**, respectively. In [^18^F]**4b** the lysine side chain is shortened by only one methylene group, which should largely conserve the physicochemical properties of the parent compound [^18^F]**4a**. Replacement of the lysine residue by ornithine in LO substrates results in attenuated turnover (Trackman and Kagan, 1979). Substitution of the amino group of the lysine side chain in [^18^F]**4a** by an inert hydrogen atom, as realized in [^18^F]**4c** by introducing a norleucine in the respective position, completely abolishes the substrate properties toward LOs. The pharmacokinetic properties of the radiotracers [^18^F]**4b** and [^18^F]**4c** were evaluated in rats with regards to their metabolic stability and whole-body biodistribution as it was performed for [^18^F]**4a**. Both analogs exhibit slightly higher metabolic stability compared to [^18^F]**4a** with 76 and 52% of original [^18^F]**4b** and [^18^F]**4c** remaining after 120 min p.i., respectively (Figure 10A), which probably reflects their decreased susceptibility toward degradation by trypsin-like serine proteases in the blood. The distribution half-lives t_1/2α_ of [^18^F]**4a** and its analogs are almost identical, which indicates that replacement of the lysine residue does not influence their tissue penetration. In accordance with their higher stability, the elimination half-lives t_1/2β_ of [^18^F]**4b** and [^18^F]**4c** were slightly greater than that of [^18^F]**4a** (Figure 10B). The whole-body biodistribution in normal Wistar rats of [^18^F]**4b** and [^18^F]**4c** was largely comparable to that of [^18^F]**4a** for both time points, 5 and 60 min p.i. (see Figures 10C–E for selected organs and Figures S9, S10 in Supplementary Material for the complete organ set), which confirmed the assumption that changes at the lysine residue of the parent compound will result in only minor alterations in the pharmacokinetic behavior. The liver uptake was slightly higher for [^18^F]**4c** than for [^18^F]**4a** and [^18^F]**4b**, which is in agreement with its more hydrophobic character due to the neutral vs. positively charged residues.

**Figure 11:**
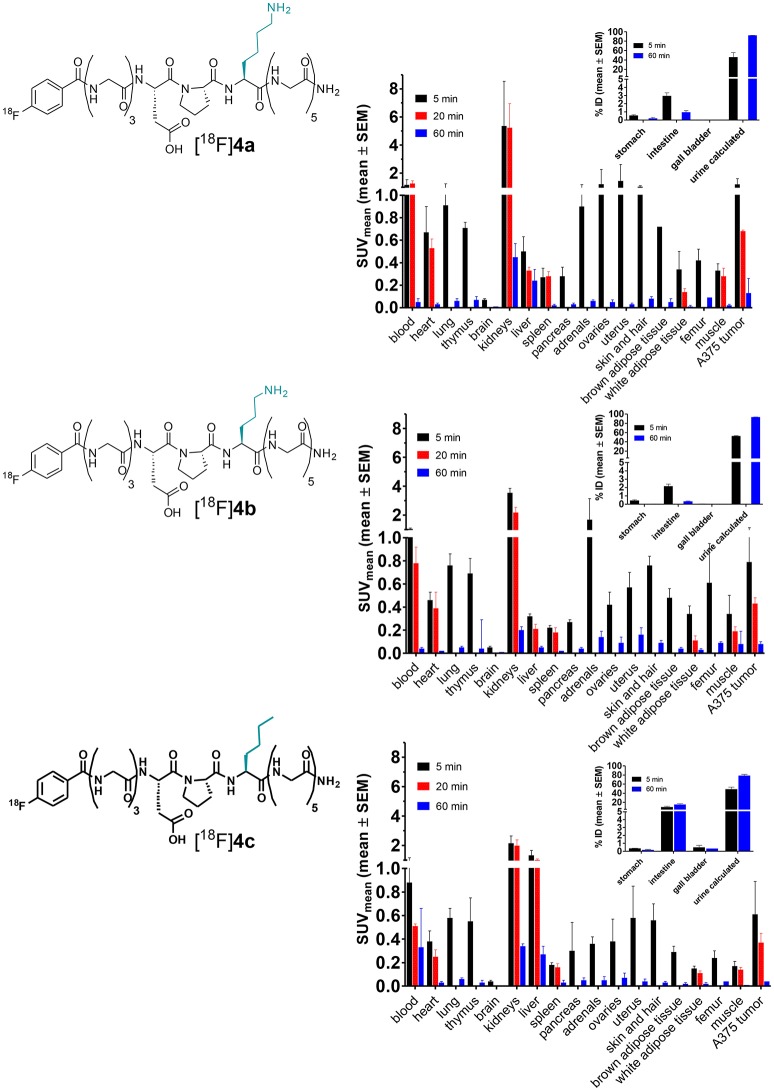
Biodistribution of [^18^F]**4a** and its ornithine ([^18^F]**4b**) and norleucine ([^18^F]**4c**) analogs in A375 melanoma-bearing NMRI nu/nu mice. The activity uptake in distinct organs is expressed as standardized uptake values (SUV; *n* = 2–3/time group). For 20 min p.i., not all organs were considered for analysis. The insets show activity uptake in distinct organs expressed as % of total injected ^18^F activity (%ID, mean ± SEM).

Comparative evaluation of the radiotracers [^18^F]**4a**-**c** in the A375 melanoma xenograft-bearing mice was performed on the basis of *ex vivo* whole-body biodistribution for the time points 5, 20, 60 min p.i. (Figure 11). As expected, the biodistribution patterns of all three compounds are similar when the results from both species are compared. However, the kidney uptake varied more between the individual tracer compounds in the mice than in the rats. The highest kidney uptake in the mice was displayed by [^18^F]**4a**, the uptakes of [^18^F]**4b** and [^18^F]**4c** in that organ were gradually lower. Apart from the differential kidney uptake, only minor variations between the biodistributions of the three radiotracers are obvious. Exceptions are the liver and the reproductive organs. Similar to the result from biodistribution studies in rats, activity accumulation in the liver is significantly more pronounced for compound [^18^F]**4c**, which probably accounts for the increased uptake in the intestine that can be observed for this radiotracer compared to [^18^F]**4a** and [^18^F]**4b**. Considering the tumor uptake of the three analogs (Table 3), the determined SUV at 5 min p.i. is greater than 1 for [^18^F]**4a**, which is in agreement with the results obtained from the dynamic PET imaging investigations. In contrast, SUV at that time point is lower than 1 for [^18^F]**4b** and [^18^F]**4c**. Furthermore, the SUVs for all three time points differ in the order [^18^F]**4a**>[^18^F]**4b**>[^18^F]**4c**. This finding is in agreement with the substrate properties of the tracer compounds toward LOs. The SUVs for the tumor region at 20 min p.i. are statistically significantly lower for [^18^F]**4b** and [^18^F]**4c** compared to [^18^F]**4a**. Notably, the uptake of [^18^F]**4b** is slightly higher than that of [^18^F]**4c**, which is likely due to the fact that the ornithine analog [^18^F]**4b** can be still recognized by LOs as substrate despite the by one methylene group shortened side chain, even though with much lower affinity compared to the lysine analog [^18^F]**4a** (Trackman and Kagan, 1979). In contrast, the capability for covalent interactions with LOs is completely abolished for the norleucine analog [^18^F]**4c**. The SUV ratio at 20 min p.i. between [^18^F]**4a** and [^18^F]**4c** is with a value of 1.84 in a range that has been obtained with negative-control analog of radiotracers for other tumor-associated targets (Haubner et al., 1999). Furthermore, the uptake ratio [^18^F]**4c**/[^18^F]**4a** suggests that about 54% of the uptake signal is contributed by non-specific, i.e., LO-independent targeting, as [^18^F]**4c** as is not capable of interacting with the target enzymes; hence, its uptake can be considered as LO-independent. The obtained results for the negative-control analogs of [^18^F]**4a** provide further evidence toward the LO-mediated uptake of this radiotracer in the melanoma xenografts.

**Table 3:** Tumor uptake of [^18^F]**4a** and its ornithine ([^18^F]**4b**) and norleucine ([^18^F]**4c**) analogs as determined by *ex vivo* biodistribution in A375 melanoma-bearing NMRI nu/nu mice shown in Figure 11 and graphical representation of the tumor uptake at 20 min p.i.

	**SUV**			
**t (min)**	**[^18^F]4a**	**[^18^F]4b**	**[^18^F]4c**	
5	1.20 ± 0.41 (*n* = 2)	0.79 ± 0.34 (*n* = 2)	0.61 ± 0.28 (*n* = 3)	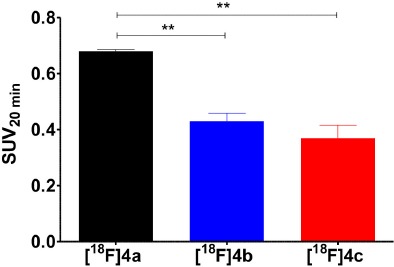
20	0.68 ± 0.01 (*n* = 3)	0.43 ± 0.05 (*n* = 3)	0.37 ± 0.08 (*n* = 3)	
60	0.13 ± 0.13 (*n* = 3)	0.08 ± 0.02 (*n* = 3)	0.04 ± 0.00 (*n* = 3)	

Data were compared with [^18^F]4a using the unpaired t test, with **indicating P < 0.01 (P = 0.0011 and 0.0026 for [^18^F]4b and [^18^F]4c, respectively).

## Conclusions

This study has demonstrated that imaging of the melanoma-associated LOs is possible with substrate-derived radiotracers using dynamic PET measurements. Even though irreversible radiotracer retention cannot be observed due to the absence of crosslinking to collagen *in vivo*, a LO-mediated tumor uptake is detectable on the basis of the temporary enzyme-substrate interaction. This conclusion is supported by the differential tumor uptake of [^18^F]**4a** and its non-functional analogs [^18^F]**4b** and [^18^F]**4c**. The A375-derived xenograft model was used exemplarily for those melanoma which are characterized by increased expression of these prometatastic enzymes.

The obtained results will advance further research activities toward the non-invasive detection of tumor-associated LO activity and stimulate efforts aiming on the development of agents capable of LO targeting for both imaging and therapeutic purposes.

Considering the heterogeneity of melanoma mentioned above, our observation represents rather a basic finding on one commonly used specific melanoma cell line than a generalizable finding with a prompt specific clinical relevance in melanoma.

## Materials and methods

### Synthesis and radiolabeling of peptides 4b and 4c

#### General remarks

All chemical reagents and solvents were obtained from commercial suppliers and used without further purification. Fmoc-protected amino acids and coupling reagents were purchased from MultiSynTech (Witten, Germany) and Iris Biotech (Marktredwitz, Germany). Fmoc-Rink-Amide MBHA polystyrene resin was obtained from Multisyntech. Mass spectra were obtained on a Quattro LC (Waters, Milford, MA, USA) or a Xevo TQ-S (Waters, Milford, MA, USA) mass spectrometer equipped with an electrospray ionization source, each driven by the Mass Lynx software.

#### Peptide synthesis

All peptides were synthesized by a microwave-assisted, fully automated solid-phase peptide synthesis (SPPS) based on the Fmoc-protecting group strategy with appropriate side chain-protected l-amino acids as building blocks using a Liberty Microwave Peptide Synthesizer (CEM, Matthews, NC, USA) combined with a Discover microwave reactor (CEM, Matthews, NC, USA). Peptides **2** and **4a** were synthesized as previously published (Kuchar et al., 2012). Syntheses of peptides **1**, **4b**, and **4c** followed the same procedure, with the exceptions that pyroglutamic acid was introduced N-terminally during automated microwave-assisted SPPS for peptide **1** and Fmoc-Lys(Boc)-OH was replaced by Fmoc-Orn(Boc)-OH and Fmoc-Nle-OH as building blocks in the synthesis of **4b** and **4c**, respectively. MS (ESI+) data for peptides **1**, **4b** and **4c**: *m*/*z*(**1**) = 862.32 ([M+2H]^2+^); *m*/*z*(**4b**) = 461.88 ([M+2H]^2+^), 922.52 ([M+H]^+^); *m*/*z*(**4c**) = 480.37 [M+K+H]^2+^, 921.55 [M+H]^+^. Full mass spectra of these peptides can be found in Supplementary Material (Figure S4). The cyclic peptide **3** was synthesized as recently published (Wodtke et al., 2015). The resin-bound, N-terminally unmodified peptides **2** and **4a**–**c** and the resin-bound peptide **3** exhibiting an unmodified side chain of the second lysine residue served as labeling precursors for reaction with [^18^F]SFB as detailed below.

#### Synthesis of *N*-succinimidyl 4-[^18^F]fluorobenzoate ([^18^F]SFB)

The automated radiosynthesis of [^18^F]SFB was performed as described in Mäding et al. (2005) with implemented modifications as published in Kapty et al. (2011) and Kuchar et al. (2012). To further increase the molar activity of [^18^F]SFB and consequently that of the radiolabeled peptides, the obtained crude [^18^F]SFB was purified by semi-preparative HPLC by the following procedure, which was integrated into the automated process. Crude [^18^F]SFB was diluted with 1.5 mL H_2_O/MeCN (65:35 v/v including 0.1% acetic acid). The filtered mixture was subjected to a semi-preparative HPLC system including pump (Jasco PU 980), UV-filterphotometer (KNAUR, 220 nm), and radioactivity detector (Nuclear Interface) and a Discovery HSF-5 column (Supelco; 250 × 10 mm, 5 μm with precolumn 4 × 9 × 4 mm). HPLC purification was carried out at a flow rate of 4 mL/min and isocratic conditions [eluent: H_2_O/MeCN 65:35 (v/v) containing 0.1% acetic acid]. The collected [^18^F]SFB-containing fraction was diluted into 30 mL H_2_O, adsorbed on HBL (500 mg, Oasis) solid-phase extraction cartridge and eluted with 2 mL MeCN. The radiochemical and chemical purity of the final product was determined on a Hewlett-Packard system consisting of binary pump (G1312A), degasser (G1322A), column oven (G1316A), data interface (Agilent Technologies 1200 Infinity universal interface box II), diode array detector (G1314D), and gamma detector (GABI, Raytest). A Kinetex 5 μ PFP 100A (150 × 4.6 mm; Phenomenex) column was used as stationary phase. Water/Acetonitrile (65:35) at a flow rate of 0.5 mL/min was used as mobile phase under isocratic conditions. Monitoring of UV absorption was done at a wavelength of 235 nm. In a typical radiosynthesis, 3 GBq of [^18^F]SFB (molar activity 60–75 GBq/μmol, radiochemical purity >99%) were yielded from 18 GBq of [^18^F]fluoride.

#### Preparation of radiolabeled peptides

The labeling of all peptides was carried out by reacting the resin-bound peptidic precursors with [^18^F]SFB (Schemes S1 and S2 in Supplementary Material) as published by Kuchar et al. (2012) with the slight modification that cleavage of the ^18^F-fluorbenzoylated peptides from resin was carried out in 300 μL trifluoroacetic acid/water/triisopropylsilane (95:4:1, v/v/v). Subsequently, the resin was filtered off and the filtrate was diluted into 500 μL of the solvent mixture that corresponded to the eluent mixture of the semi-preparative HPLC. For each peptide, conditions for analytical and semi-preparative HPLC were elaborated separately and are specified in Supplementary Material (Tables S2, S3, S5). Radiochemical parameters for the synthesis of the ^18^F-labeled peptides are included in Table S4 in Supplementary Material. (Radio-)HPLC chromatograms can be found in Figures S5 and S6 in Supplementary Material.

### Determination of binding affinity to collagen by SPR

In order to probe binding of the telopeptide derivatives to atelocollagen surface plasmon resonance (SPR) measurements were performed on a T100 Biacore system (GE Healthcare) (Hoppmann et al., 2010). In brief, bovine type I atelocollagen (=pepsin-treated type I collagen; Matrix Bioscience) was immobilized as ligand on a CM5 (carboxymethylated dextran polymer) sensor chip using EDC/NHS chemistry. About 12,000 arbitrary response (resonance) units (RU) were immobilized. Various concentrations of the analytes (**1**, **2**, and **4a**) ranging from 45 to 950 μM were analyzed. The flow buffer used was 10 mM HEPES, 3.0 mM EDTA-Na_2_, 0.15 M NaCl (pH 7.4) containing 0.05% surfactant P20. Association and dissociation of all analytes was followed in real-time and measured at 25°C at a flow rate of 30 μL/min. After each cycle, the surface was regenerated using 50 mM NaOH. The data were fitted to a two-state reaction model (see scheme below) including reference subtraction and blank buffer correction derived from blank runs using the Biacore Evaluation software 2.0.4.

$$\text{R}+\text{L}\underset{k_{d1}}{\overset{k_{a1}}{⇌}}\text{RL}\underset{k_{d2}}{\overset{k_{a2}}{⇌}}{\text{RL}}^{\text{*}}$$

The parameters *K*_D_, *k*_off_, and *k*_on_ were calculated from the individual rate constants by the following equations (Tummino and Copeland, 2008):

(1) $$K_{D}=\frac{k_{d1}}{k_{a1}+\frac{k_{a1}k_{a2}}{k_{d2}}}$$

(2) $$k_{off}=\frac{k_{d1}k_{d2}}{k_{a2}+k_{d1}+k_{d2}}$$

(3) $$k_{on}=\frac{k_{off}}{K_{D}}$$

### Cell culture

The human melanoma cell lines A375, A2058, MeWo (purchased from ATCC, CRL-1619, CRL-1147, HTB-65), MEL-JUSO (purchased from DSMZ, ACC-74) were cultured and cell extracts prepared thereof as published elsewhere (Oswald et al., 2007; Reissenweber et al., 2013).

### Protein expression analysis

For the analysis of secreted proteins, the medium supernatant was collected and transferred to an Eppendorf tube after a cultivation time of 24 h, and centrifuged for 15 min at 16,000 g and 4°C. Subsequently, the supernatant was discarded, apart from 40 μL, which remained in the tube and were stored at −65°C.

For the analysis of cell-associated proteins, the cell sediment was washed twice with PBS. RIPA lysis buffer (200 μL per well) was added and incubated for 15 min on ice. The cell suspension was transferred to an Eppendorf tube, sheared by passage through a cannula (diameter 0.5 mm) and ultrasonicated for 10 s. The cell fragments were removed by centrifugation for 10 min at 10,000 g and 4°C. The protein-containing supernatant was transferred and stored at −65°C.

Isolation of proteins from tumor and other tissues was performed by immersing the tissue sample in RIPA buffer (400 μL per 100 mg of tissue) in a 2 mL Eppendorf tube (not more than 200 mg of tissue per tube) followed by sonification [Bandelin electronics UW 2200; 180 s at 25 Hz, 15 s at ultrasound (20%)] and 20 min of incubation in the tumble shaker (polymax 1040, Heidolph, Germany). The tissue fragments were removed by centrifugation for 20 min at 12,000 g and 4°C. The protein-containing supernatant was transferred and stored at −65°C.

Determination of protein concentration was performed by using the commercially available Pierce BCA protein assay kit (Thermo Scientific). The concentration was determined by measuring the absorption at 562 nm using a calibration curve obtained from standard solutions of bovine serum albumin of defined concentration.

For separation by SDS-PAGE, the protein samples were incubated with SDS sample buffer (7–10 μL) at 99°C for 5 min. Thirty to fifty microliters of the resulting solution (corresponding to 10–75 μg protein) were loaded to the gel. In addition, 5 μL of the molecular mass marker (PageRuler^TM^ plus prestained protein ladder) were loaded to a separate lane. SDS-PAGE was performed at constant voltage in a BioRad apparatus. Subsequently, proteins were transferred from the gel to nitrocellulose (LOX) or PVDF (LOXL2) membranes by electroblotting (BioRad Trans-Blot Turbo transfer system; constant voltage) and blocked for 60–90 min with a solution of non-fat dry milk powder (5%, w/v) in Tris-buffered saline containing 0.05% (v/v) Tween 20 (TBS-T). For detection of LOX and LOXL-2, membranes were incubated with the following primary antibodies: LOX H140 (1:750, catalog number sc-66947; Santa Cruz Biotechnology), LOXL2 AF2639 (1:200, R&D) and rabbit anti-actin (1:1,000, catalog number A5420; Sigma-Aldrich) followed by incubation with peroxidase-conjugated goat anti-rabbit IgG secondary antibodies (1:10,000; Sigma- Aldrich). Incubation with the primary and secondary antibodies were performed in TBS-T; 2 h at room temperature and overnight at 4°C for primary antibodies, washed with TBS-T (3 × 10 min) and TBS (10 min); 90 min at room temperature for secondary antibody followed by washing with TBS-T (3 × 10 min). Semiquantitative densitometric evaluation of Western blot bands was performed using the AIDA software (Raytest, Straubenhardt, Germany). Expression ratios relative to actin were calculated from the profile peak areas determined under identical quantum level range settings for the lysyl oxidase and actin control bands.

### Radiopharmacological methods

Experiments were performed in accordance with the guidelines of the German Regulations for Animal Welfare after approval by the local Ethics Committee for Animal Experiments appointed by the State Authority of Saxony (Landesdirektion Sachsen, Stauffenbergallee 2, 01099 Dresden, Germany; reference numbers 24-9168.11-4/2012-1 “Untersuchungen zur Bedeutung von Rezeptor-Ligand-Wechselwirkungen bei der Tumorprogression und Metastasierung des malignen Melanoms,” 24-9168.21-4/2004-12 “Nachweis der Unbedenklichkeit neuer radioaktiver Stoffe vor ihrer ersten Anwendung am Menschen—Untersuchung von Verteilung, Metabolismus und Wechselwirkungen zur Bewertung des Einflusses individueller Faktoren auf die Verteilungseigenschaften von potentiellen Radiopharmaka im Organismus” and 4D-9168.11-4/2007-2 “Untersuchungen zur Bedeutung ausgewählter Protein-Protein-Wechselwirkungen für Metastasierung und Tumorprogression im modifizierten syngenen B16-Melanom-Modell”).

For the investigation of metabolic stability, 20–30 MBq of the respective radiotracer dissolved in physiological saline (0.5 mL) were injected into the tail vein of male Wistar rats under anesthesia with a gas mixture of 10% desflurane and 30% oxygen/air. At defined time points blood samples were taken from right femoral artery via catheter. The exact volume and activity (decay-corrected according to time of injection) of the blood samples was determined for SUV calculation (Equation 5). The samples were centrifuged at 3 min at 11,000 g, the obtained supernatant was removed and treated with ice-cold methanol for precipitation of plasma proteins. The resulting suspension was centrifuged again (11,000 g, 3 min) and subjected to HPLC analysis on an Agilent 1100 system equipped with UV/Vis DAD and a radiation detector (Canberra-Packard, Radiomatic Flo-one Beta 150TR—with PET flow cell). A Zorbax 300SB-C18 column (250 × 9.4 mm; 5 μm) was used as stationary phase, elution was done in gradient mode at a flow rate of 3 mL/min using the following programme: 0–5 min 95% water/5% acetonitrile/0.1% trifluoroacetic acid, 5–15 min gradient 100% acetonitrile/0.1%trifluoroacetic acid, 15–20 min plateau at 100% acetonitrile/0.1% trifluoroacetic acid, 20–23 min back to 0–5 min 95% water/5% acetonitrile/0.1% trifluoroacetic acid.

The ratio of area under the curve of the original compound to the summed peak areas of all observed species was calculated for every time point. The obtained fractions were multiplicated with the SUV of the corresponding blood samples. The calculated values were plotted against the time and analyzed by Equation (4) describing drug elimination in a two-compartment model:

(4) $$\begin{array}{l} \;A=A_{1}e^{-k_{\alpha }t}+A_{2}e^{-k_{\beta }t}+plateau\\ \;A=measured\;activity\;(SUV)\\ k_{\alpha }=\frac{\ln2}{t_{1/2\alpha }},k_{\beta }=\frac{\ln2}{t_{1/2\beta }} \end{array}$$

Determination of the whole body biodistribution of the radiotracers [^18^F]**2**, [^18^F]**3**, [^18^F]**4a**-**c** in healthy male Wistar rats (aged 6–8 weeks, 140–185 g body weight; *n* = 4–12 per time point and radiotracer) was done by injecting 1.2–2.0 MBq of the respective radiotracer dissolved in physiological saline (0.5 mL) into the lateral tail vein of male Wistar rats. After 5, 20 (mice only), and 60 min, the animals were anesthetized and sacrificed by cardiac punction. All major organs including glands, adipose and muscle tissue were collected, and their mass and activity were determined; furthermore, the mass and activity of tumors (mice only) the skin, blood, and the remaining body were determined. Activity was counted in a Wallac WIZARD automatic γ-counter (PerkinElmer, Germany). The radioactivity of the tissue samples was decay-corrected and calibrated by comparing the counts in tissue with the counts in aliquots of the injected tracer that had been measured in the γ-counter at the same time. The SUVs for the individual organs were calculated according to Equation (5).

(5) $$\begin{array}{l} SUV=\frac{activity\;concentration\;of\;X\;\left(\frac{Bq}{g}\right)}{A_{Start}(Bq)}*mass\;of\;X(g)\\ \;X=organ,\;tissue\;or\;ROI\\ \;A=injected\;start\;activity \end{array}$$

The A375 melanoma xenograft model was generated as described previously (Mamat et al., 2012). Cultured A375 melanoma cells were harvested, washed in PBS, and transferred to 0.9% NaCl. For each animal, 5 × 10^6^ cells in 100 μl were prepared for injection. Twelve to fourteen weeks old female NMRI (nu/nu) mice were purchased from the specific pathogen-free breeding facility of the Experimental Centre of the Medical Faculty Carl Gustav Carus, University of Technology, Dresden. Animals were anesthetized using desflurane (10% v/v in 30% oxygen/air), and melanoma cells were subcutaneously injected into the right hind leg. Tumor size was monitored thrice weekly by caliper measurements, and tumor volume was calculated using the formula V = π/6 × (tumor length × tumor width × tumor width). Animals entered the study 20–27 days after tumor cell injection with tumor volumes between 100 and 800 mm^3^.

For whole-body radioluminography of [^18^F]**2**, [^18^F]**3**, [^18^F]**4a** [here 20–30 MBq of the respective radiotracer dissolved in physiological saline (0.2 mL) were injected], the A375-bearing mice were deeply anesthetized and sacrificed using carbon dioxide inhalation 60 min after radiotracer injection and frozen in liquid nitrogen. Subsequently, the bodies were embedded in aqueous carboxymethylcellulose and frozen in liquid nitrogen. The frozen block was mounted onto a microtome (Jung cryopolycut) and cutted automatically until the desired sectional plane was reached. Then, the sections were prepared manually in a thickness of 70–80 μm at −16°C. The sections were mounted to a holder and freeze-dried at −10°C and 14 mbar for 1 h (Sublimator 400, Zirbus Technology) and exposed to a phosphor imaging plate for 80 min. and scanned in a BioImaging Analyzer BAS-5000 (Fuji Photo Film) (Mamat et al., 2012).

Dynamic small animal PET imaging experiments for the radiotracers were performed in healthy male Wistar rats and A375 tumor-bearing NMRI (nu/nu) mice. Anesthetized, spontaneously breathing animals were allowed to stabilize for 10 min after preparation. A 10 min transmission scan was recorded during this time for each subject by using a rotating point source of ^57^Co (microPET P4; Siemens preclinical solutions, Knoxville, TN) or a whole-body CT was recorded (NanoScan PET/CT; Mediso Budapest, Hungary). The transmission scans were used to correct the emission scan for γ-ray attenuation caused by body tissues and supporting structures; they were also used to demarcate the body field for image registration. The activity of the injection solution was measured in a well counter (Isomed 2000, Dresden, Germany) cross-calibrated to the small-animal PET scanners. The PET acquisition of 60- or 120 min emission scans was started and the infusion of the respective radiotracer into a tail vein was initiated with a delay of 30 s. At the end of the experiment, the animals were deeply anesthetized and sacrificed by an intravenous injection of potassium chloride.

In the PET experiments, 10–20 MBq of the respective radiotracer dissolved in physiological saline (rats 0.5 mL, mice 0.2 mL) were administered intravenously as an infusion (1 mL/min) with a syringe pump (Harvard Apparatus) using a needle catheter into a tail vein.

Transmission scans and PET acquisition followed the protocol given by us in detail elsewhere (Kniess et al., 2012) The standardized uptake values (SUV) were calculated over the region of interest (ROI) as the ratio of activity concentration at time t and injected dose at the time of injection divided by body weight. ROI definition was performed using the ROVER software, version 3.0.29 (ABX GmbH, Radeberg, Germany). Summed frames from 30 to 60 min post injection (p.i.) were used to define the ROIs. The ROIs (3D) were located over the tumor and the muscle of contralateral hindleg and the heart (blood pool). After PET scan, animals were sacrificed and subjected to whole-body radioluminography as described above or to tumor resection (Mamat et al., 2012).

## Author contributions

RL: conceptualized research and wrote the manuscript, which has been reviewed and approved by all authors; MK, CN, RB, JL, and RW: performed experiments and analyzed data; BB, TK, JS, JP, and RL: supervised research; TK, JS, and JP: provided resources; JS, JP, and RL: acquired funding.

### Conflict of interest statement

The authors declare that the research was conducted in the absence of any commercial or financial relationships that could be construed as a potential conflict of interest.

## Footnotes

^1^Association of Population-based Cancer Registries in Germany [Gesellschaft der epidemiologischen Krebsregister in Deutschland e.V., Atlas der Krebsinzidenz und –mortalität in Deutschland (GEKID-Atlas)]. Available online at: http://www.gekid.de (Accessed February 12, 2018).
