# Supplementary material for: Evaluation of Fluorine-18-Labeled α1(I)-N-Telopeptide Analogs as Substrate-Based Radiotracers for PET Imaging of Melanoma-Associated Lysyl Oxidase
Source: Front Chem. 2018 Apr 26;6:121. doi: 10.3389/fchem.2018.00121 (PMC5932954; doi:10.3389/fchem.2018.00121)
Supplement: Supplementary file 1 [file Data_Sheet_1.pdf]

Supplementary Material

Evaluation of fluorine-18-labelled α1(I)-N-telopeptide analogues as substrate-based radiotracers for PET imaging of melanoma-associated lysyl oxidase

Manuela Kuchar, Christin Neuber, Birgit Belter, Ralf Bergmann, Jens Lenk, Robert Wodtke, Torsten Kniess, Jörg Steinbach, Jens Pietzsch, Reik Löser*

*** Correspondence:** r.loeser@hzdr.de

Content **Page**

Content	Page
Figure S1. SPR sensograms for telopeptide derivatives **1**, **2**, and **4a** with immobilised bovine atelocollagen	S2
Figure S2. Lane profiles for LOX Western blot analysis	S3
Table S1. Expression ratios for LOX and LOXL2	S3
Figure S3. Immunohistochemical detection of LOX and LOXL2 in A375 tumour sections	S4
Scheme S1. Synthesis of [^{18}F]**3**	S5
Scheme S2. Synthesis of [^{18}F]**4b** and [^{18}F]**4c**	S5
Figure S4. Mass spectra (ESI) of peptides **1**, **4b** and **4c**	S6
Table S2. Gradient elution programme for semi-preparative HPLC of [^{18}F]**3**	S7
Table S3. Gradient elution programme for semi-preparative HPLC of [^{18}F]**4b** and [^{18}F]**4c**	S7
Table S4. Parameters for radiochemical synthesis of [^{18}F]**2**, [^{18}F]**3**, [^{18}F]**4a-c**	S7
Figure S5. Analytical (radio-)HPLC chromatograms of crude [^{18}F]**3**, [^{18}F]**4b** and [^{18}F]**4**	S8
Figure S6. Analytical (radio-)HPLC chromatograms of [^{18}F]**3**, [^{18}F]**4b** and [^{18}F]**4c**	S8
Table S5. Gradient elution programme for the analytical HPLC of [^{18}F]**3**, [^{18}F]**4b** and [^{18}F]**4c**	S8
Figure S7. Biodistribution (SUV) of [^{18}F]**2**, [^{18}F]**3** and [^{18}F]**4a** in male Wistar rats	S9
Figure S8. Biodistribution (%ID) of [^{18}F]**2**, [^{18}F]**3** and [^{18}F]**4a** in male Wistar rats	S9
Figure S9. Biodistribution (SUV) of [^{18}F]**4a**, [^{18}F]**4b** and [^{18}F]**4a** in male Wistar rats	S10
Figure S10. Biodistribution (%ID) of [^{18}F]**4a**, [^{18}F]**4b** and [^{18}F]**4a** in male Wistar rats	S10

A

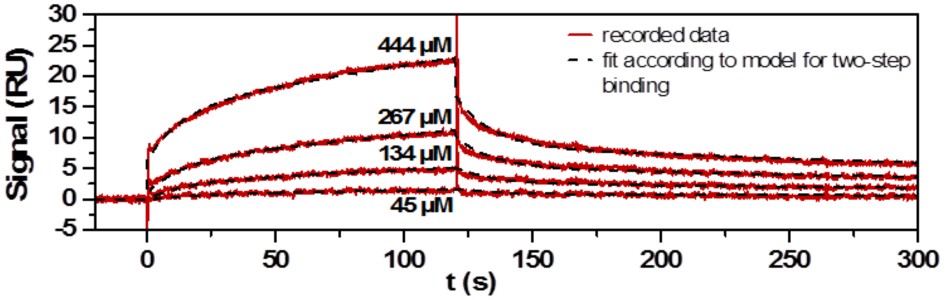

B

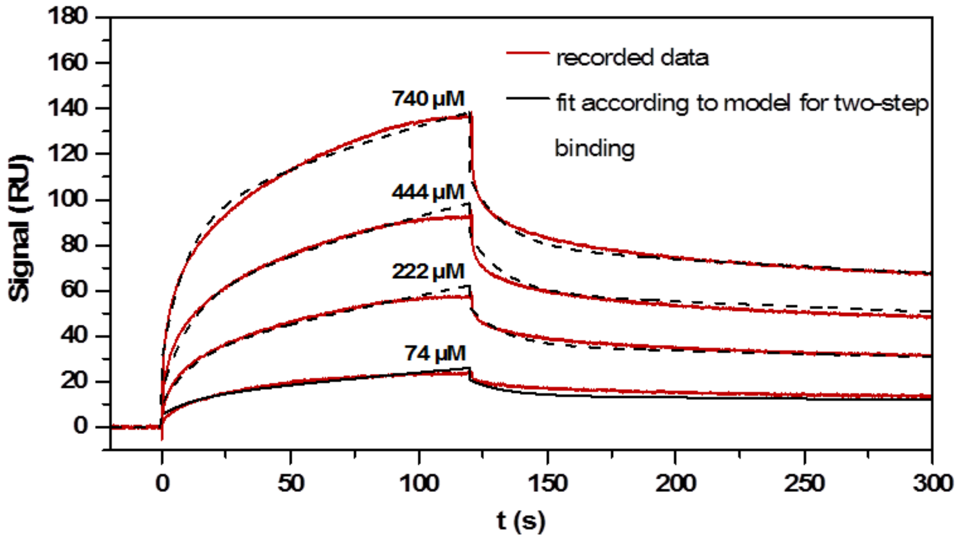

C

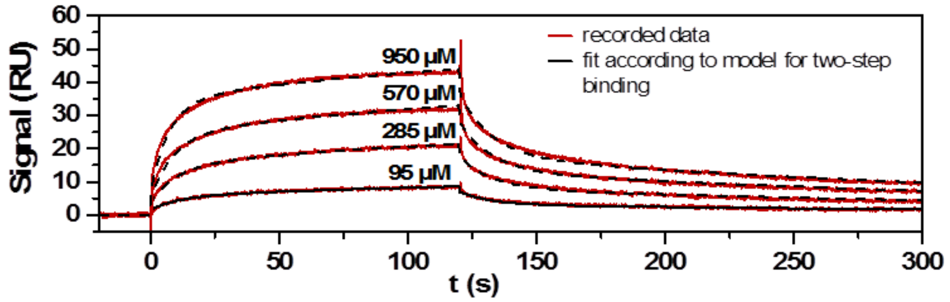

Figure S1. Interaction of telopeptide derivatives **1** (A), **2** (B), and **4a** (C) with immobilised bovine atelocollagen as investigated by surface plasmon resonance binding experiments. Closed circles represent observed data; red solid lines indicate computational fits derived from a two-step reaction model.

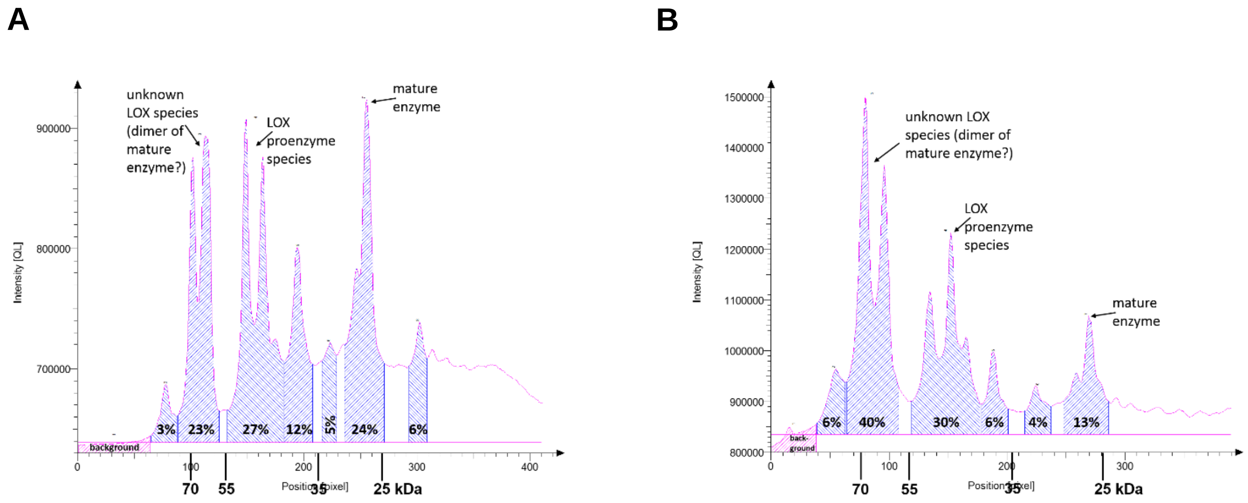

Figure S2. Lane profiles for LOX Western blot analysis of A375 cell lysates (**A**; corresponding to lane 1 in Figure 2) and supernatants (**B**; corresponding to lane 2 in Figure 2 in main text). Selected molar mass bars were attached to the pixel scale for orientation. For semiquantitative information, relative peak areas rounded to integral values are shown. The origin of the multiple LOX protein bands is discussed in the main text.

Table S1. Expression ratios for lysyl oxidase isoforms LOX and LOXL2 calculated from Western blot analyses shown in Figure 2 (expression in relation to actin; data taken from lanes 1 and 3) and Figure 3A and B (expression ratios between tumour tissue and isolated A375 cells).

	**Expression ratio LO/actin in A375 cell lysates**	**Expression ratio A375 tumour tissue/cells**[b]
**LOX**[a]	3.2	1.3
**LOXL2**	4.9	2.0

[a]Only the bands around 25 kDa corresponding to the mature enzyme were considered for calculation.
[b]Calculated from the target protein/actin ratio in tumour tissue and cells, respectively.

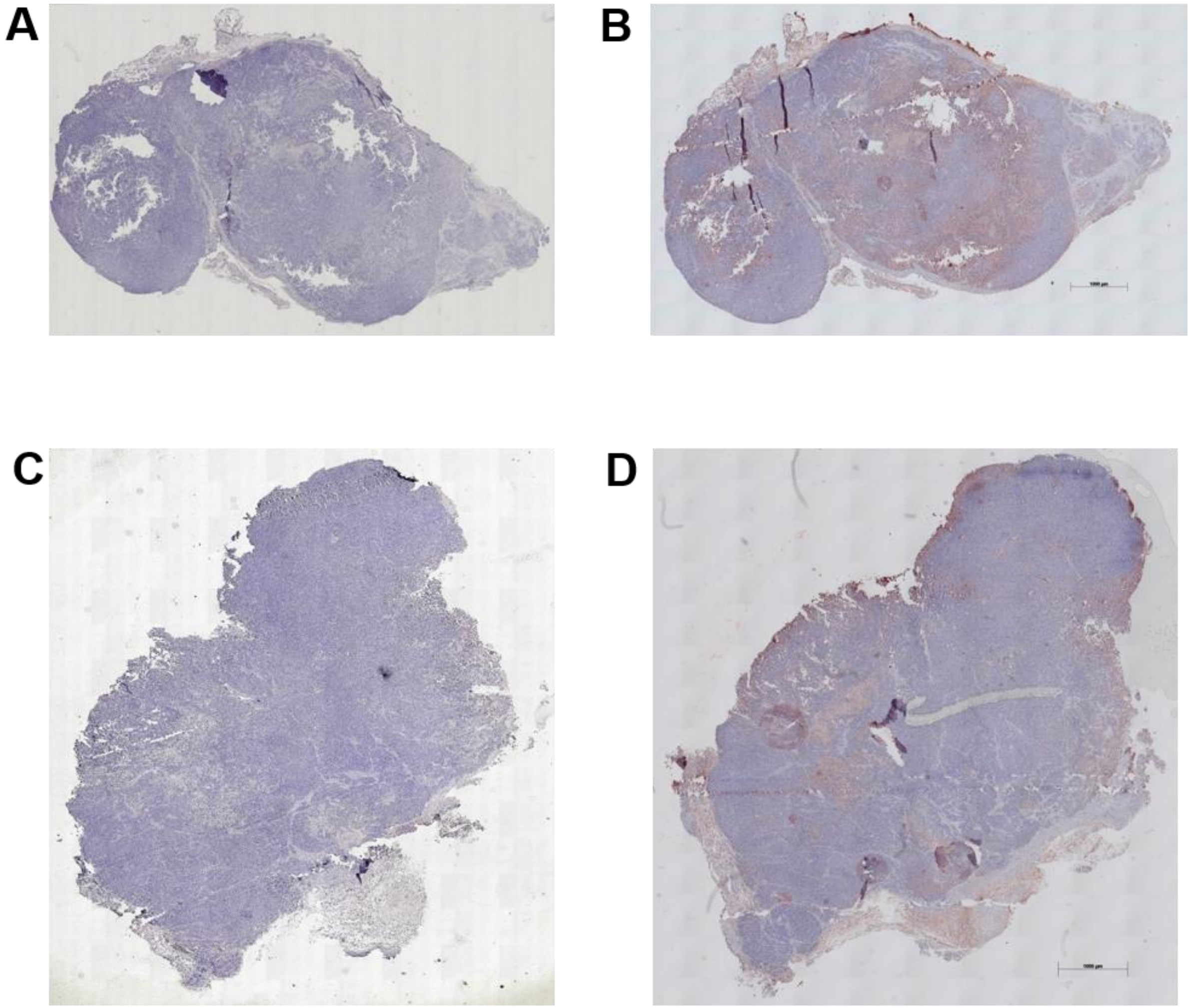

Figure S3. Immunohistochemical detection of LOX (A and B) and LOXL2 (C and D) in A375 tumour sections. A) and C) negative control by omitting the primary antibody, B) and D) immunostaining using AEC substrate (red-brown colour)

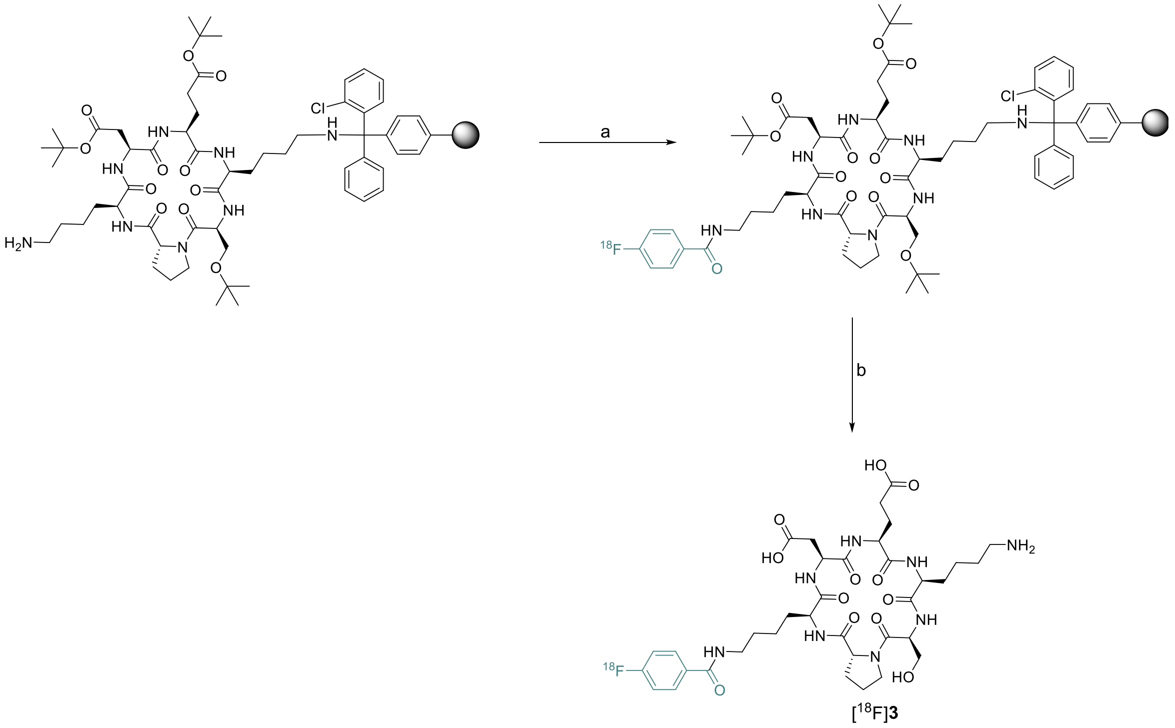

Scheme S1. Synthesis of [^{18}F]**3**. Reagents and conditions: a) [^{18}F]SFB, DMF/aqueous 0.1 M sodium phosphate pH 7.0 (3:1, v/v), 50 °C, 30 min; b) trifluoroacetic acid/water/triisopropylsilane (95:4:1, v/v/v), 50 °C, 20 min.

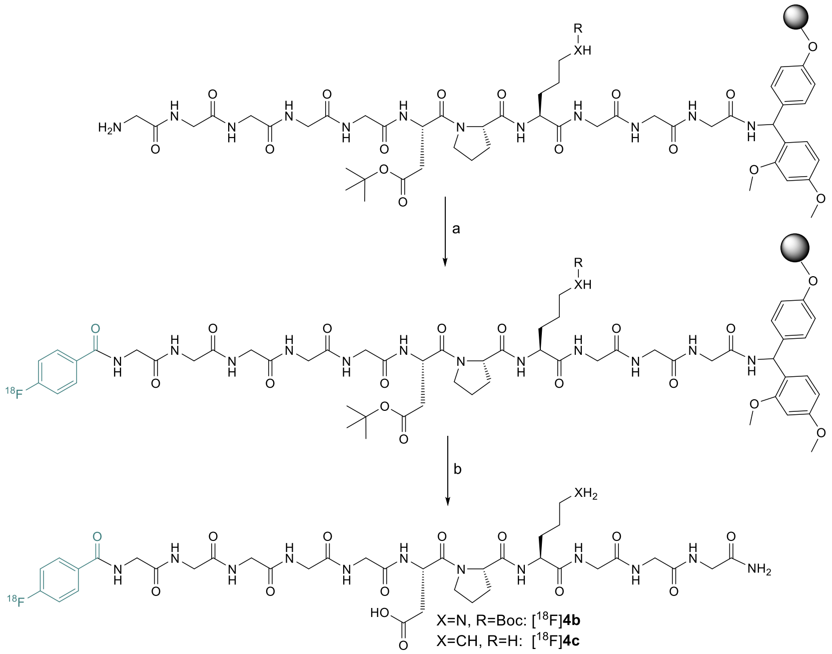

Scheme S2. Synthesis of [^{18}F]**4b** and [^{18}F]**4c**. Reagents and conditions**:** a) [^{18}F]SFB, DMF/aqueous 0.1 M sodium phosphate pH 7.0 (3:1, v/v), 50 °C, 30 min; b) trifluoroacetic acid/water/triisopropylsilane (95:4:1, v/v/v), 50 °C, 20 min.

A

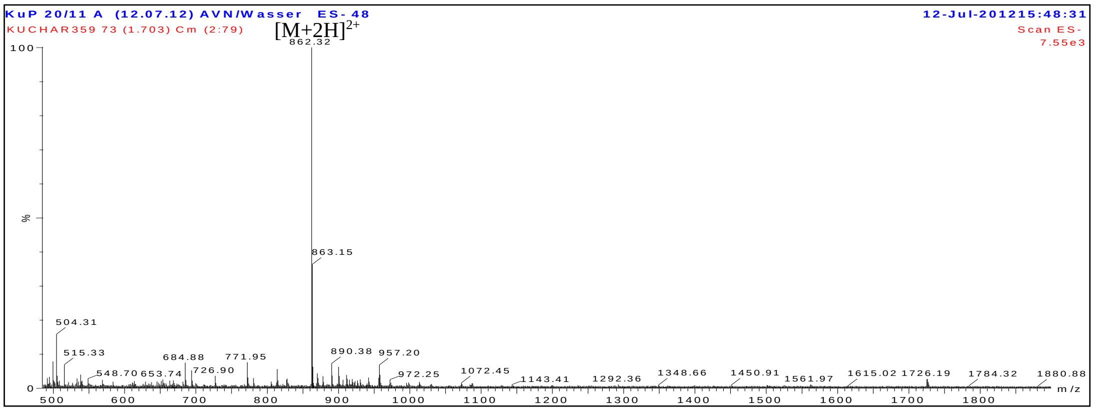

B

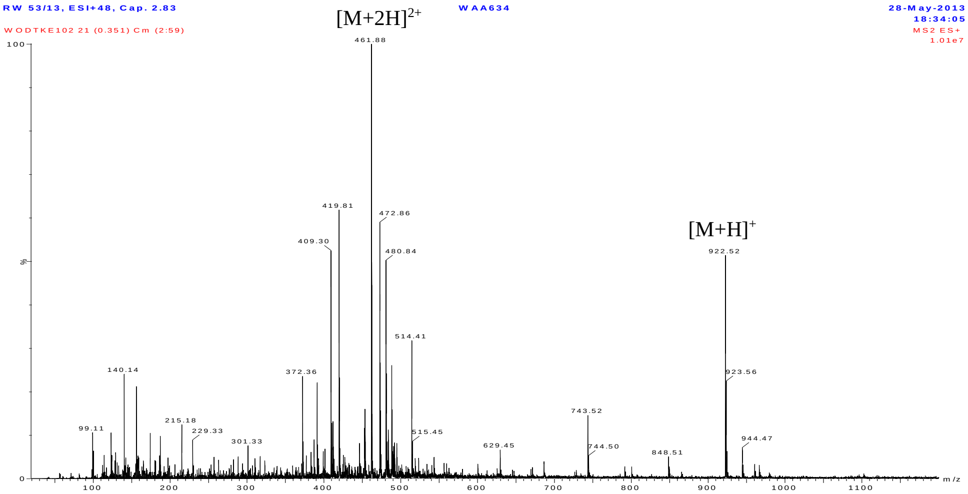

C

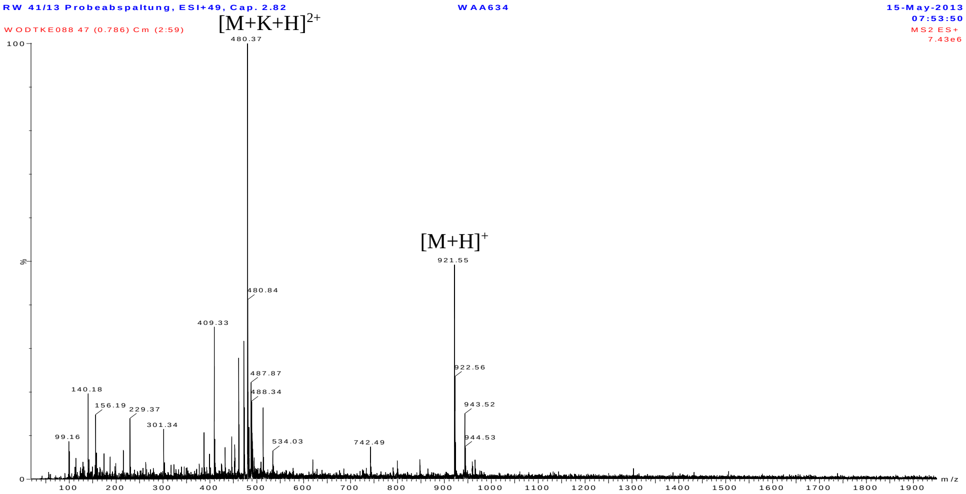

Figure S4. Mass spectra (ESI) of peptides **1** (A), **4b** (B) and **4c** (C).

Table S2. Gradient elution programme for the semi-preparative HPLC employed for purification of ^{18}F-labelled peptide [^{18}F]**3**. Separation was performed on a Jasco system (PC-driven by the Chrompass software) consisting of pump (PU-2080), degasser (DG-2080-53), data interface (LC-Net II/ADC), diode array detector (RS-232), gamma detector (Gabi, Raytest). A Macherey-Nagel VP Nucleosil 100-5 C18 (250 × 16 mm) column was used as stationary phase. The flow rate of the mobile phase was 4 mL/min, its composition is outlined below.

time (min)	H_2O + 0.1% TFA (%)	acetonitrile + 0.1% TFA (%)
0 - 3	90	10
3 - 35	65	35
35 - 40	0	100
40 - 45	0	100
45 - 50	90	10

Table S3. Gradient elution programme for the semi-preparative HPLC employed for purification of ^{18}F-labelled peptides [^{18}F]**4b** and [^{18}F]**4c**. Separation was performed on a Hewlett-Packard system consisting of quaternary pump (G1311A), degasser (G1322A), column oven (G1316A), data interface (35900), diode array detector (G1314D) and gamma detector (Gabi, Raytest). A Macherey-Nagel VP Nucleosil 100-5 C18 (250 × 16 mm) column was used as stationary phase. The flow rate of the mobile phase was 2 mL/min, its composition is outlined below.

[^{18}F]4b			[^{18}F]4c		
time (min)	water + 0.1% TFA (%)	acetonitrile + 0.1% TFA (%)	time (min)	water + 0.1% TFA (%)	acetonitrile + 0.1% TFA (%)
**0-5**	95	5	0-5	85	15
**5-20**	50	50	5-20	45	55
**20-50**	50	50	20-40	45	55
**50-55**	5	95	40-45	5	95
**55-65**	5	95	45-55	5	95
**65-70**	95	5	55-60	85	15

Table S4. Parameters for radiochemical synthesis of [^{18}F]**2**, [^{18}F]**3**, [^{18}F]**4a-c**

Product	[^{18}F]2	[^{18}F]3	[^{18}F]4a	[^{18}F]4b	[^{18}F]4c
**Radiochemical yield d.c. (%)**	18 ± 4	20 ± 5	40 ± 20	41 ± 5	52 ± 12
**Maximum of obtained activity (MBq)**	200	400	650	497	505
**Radiochemical purity (%)**	97-99	99	98-99	99	99
**Molar activity (GBq/µmol)**	1-3	1-3	1-21	31-33	16-17
**Time of synthesis (min)**	120	125	110	115	117
**n**	26	11	50	4	5

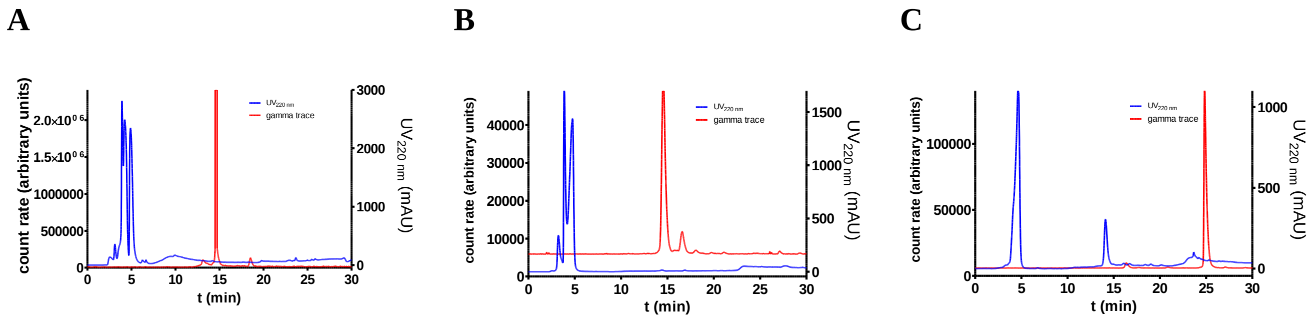

Figure S5. Analytical (radio-)HPLC chromatograms of crude ^{18}F-labelled peptides [^{18}F]**3**, [^{18}F]**4b** and [^{18}F]**4c** as obtained after cleavage from resin. A) [^{18}F]**3**, B) [^{18}F]**4b**, C) [^{18}F]**4c**.

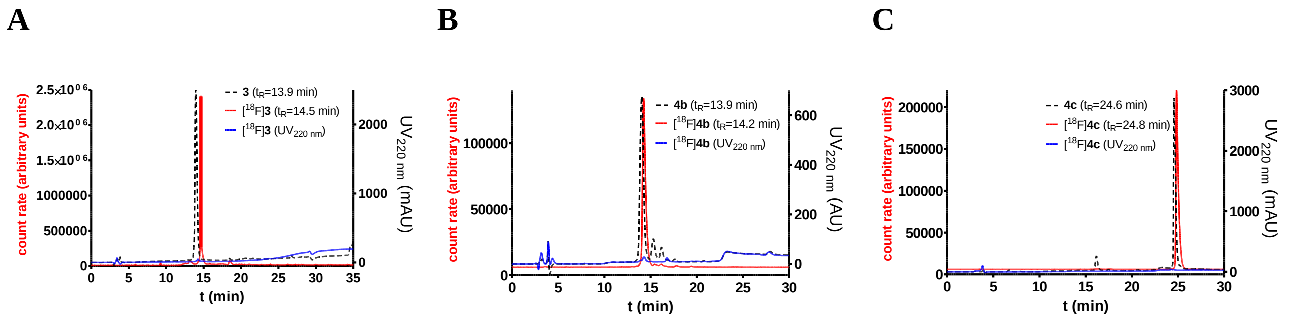

Figure S6. Analytical (radio-)HPLC chromatograms of purified ^{18}F-labelled peptides [^{18}F]**3**, [^{18}F]**4b** and [^{18}F]**4c**. For comparison, chromatograms of the corresponding non-radioactive reference compounds obtained from separate runs are overlayed. A) **3**/[^{18}F]**3**, B) **4b**/[^{18}F]**4b**, C) **4c**/[^{18}F]**4c**.

Table S5. Gradient elution programme for the analytical HPLC of ^{18}F-labelled peptides peptides [^{18}F]**3**, [^{18}F]**4b** and [^{18}F]**4c**. Analysis was performed on an Agilent Technologies 1200 system consisting of quaternary pump (G1311A), degasser (G1322A), autosampler (G1329A), column oven (G1316A), data interface (35900E), diode array detector (G1315D) and gamma detector (Gabi, Raytest). A Macherey-Nagel EC Nucleosil standard 100-7 C18 (250 × 4.6 mm) or Phenomenex Luna 5 µ C18 100A (250 × 4.6 mm) column was used as stationary phase for the purification of [^{18}F]**3** and [^{18}F]**4b**/**c**, respectively. The flow rate of the mobile phase was 1 mL/min, its composition is outlined below.

**3**			**4b** and **4c**		
time (min)	water + 0.1% TFA (%)	acetonitrile + 0.1% TFA (%)	time (min)	water + 0.1% TFA (%)	acetonitrile + 0.1% TFA [illegible]
0 - 3	90	10	0-5	90	10
3 - 35	65	35	5 - 18	82	18
35 - 40	0	100	18 - 23	50	50
40 - 45	0	100	23 - 28	50	50
45 - 50	90	10	28 - 35	30	70
			35 - 40	90	10

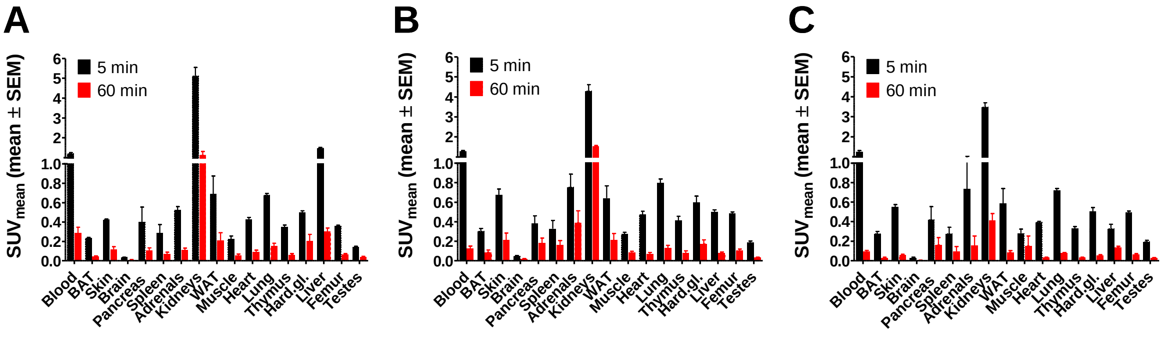

Figure S7. Biodistribution of [^{18}F]**2** (A; n=12), [^{18}F]**3** (B; n=12) and [^{18}F]**4a** (C; n=8) in male Wistar rats 5 and 60 min after a single injection. The activity uptake in distinct organs is expressed as standardised uptake values (SUV).

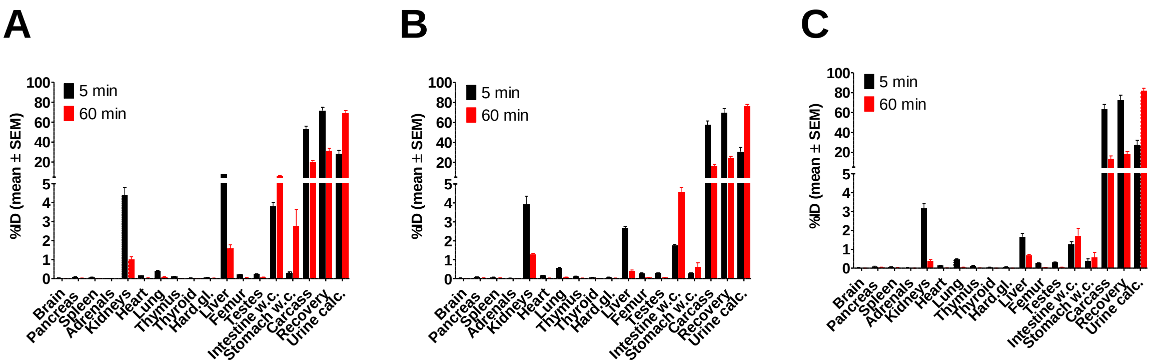

Figure S8. Biodistribution of [^{18}F]**2** (A; n=12), [^{18}F]**3** (B; n=12) and [^{18}F]**4a** (C; n=8) in male Wistar rats 5 and 60 min after a single injection. The activity uptake in distinct organs is expressed as % of total injected ^{18}F activity (%ID).

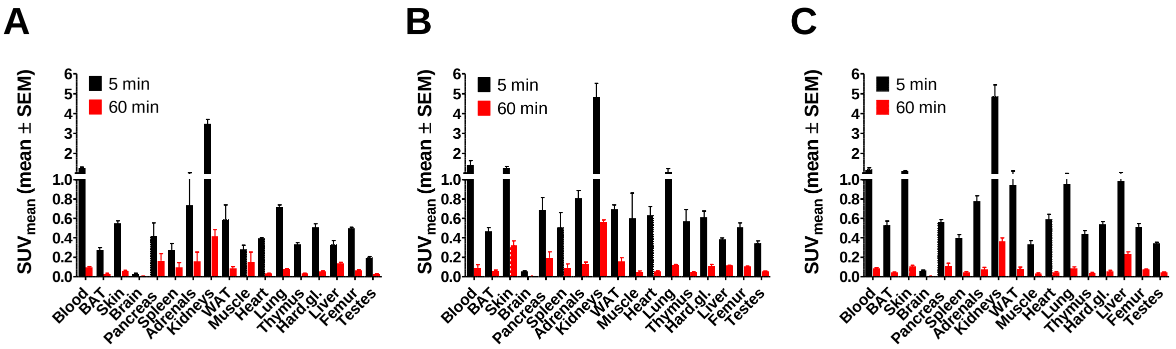

Figure S9. Biodistribution of [^{18}F]**4a** (A; n=8), [^{18}F]**4b** (B; n=4) and [^{18}F]**4c** (C; n=4) in male Wistar rats 5 and 60 min after a single injection. For comparison, the data for [^{18}F]**4a** (identical to Figure S6C) have been included. The activity uptake in distinct organs is expressed as standardised uptake values (SUV).

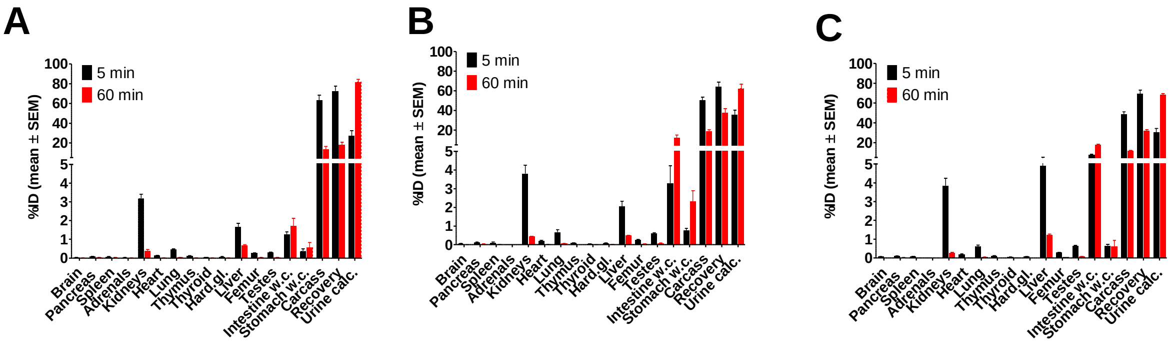

Figure S10. Biodistribution of [^{18}F]**4a** (A; n=8), [^{18}F]**4b** (B; n=4) and [^{18}F]**4c** (C; n=4) in male Wistar rats 5 and 60 min after a single injection. For comparison, the data for [^{18}F]**4a** (identical to Figure S7C) have been included. The activity uptake in distinct organs is expressed as % of total injected ^{18}F activity (%ID).
